# A diuranium carbide cluster stabilized inside a C_80_ fullerene cage

**DOI:** 10.1038/s41467-018-05210-8

**Published:** 2018-07-16

**Authors:** Xingxing Zhang, Wanlu Li, Lai Feng, Xin Chen, Andreas Hansen, Stefan Grimme, Skye Fortier, Dumitru-Claudiu Sergentu, Thomas J. Duignan, Jochen Autschbach, Shuao Wang, Yaofeng Wang, Giorgios Velkos, Alexey A. Popov, Nabi Aghdassi, Steffen Duhm, Xiaohong Li, Jun Li, Luis Echegoyen, W. H. Eugen Schwarz, Ning Chen

**Affiliations:** 1 Laboratory of Advanced Optoelectronic Materials, College of Chemistry, Chemical Engineering and Materials Science, Soochow University, Suzhou, Jiangsu 215123 China; 2 Department of Chemistry and Key Laboratory of Organic Optoelectronics & Molecular Engineering of the Ministry of Education, Tsinghua University, Beijing, 100084 China; 3 Soochow Institute for Energy and Materials InnovationS (SIEMIS), College of Physics, Optoelectronics and Energy & Collaborative, Soochow University, Suzhou, Jiangsu 215006 China; 4 Mulliken Center for Theoretical Chemistry, Universität Bonn, 53115 Bonn, Germany; 5 Department of Chemistry, University of Texas at El Paso, 500 West University Avenue, El Paso, TX 79968 USA; 6 Department of Chemistry, University at Buffalo, State University of New York, Buffalo, NY 14260-3000 USA; 7 School of Radiological and Interdisciplinary Sciences & Collaborative Innovation Center of Radiation Medicine of Jiangsu, Higher Education Institutions, Soochow University, Suzhou, Jiangsu 215123 China; 8 Nanoscale Chemistry, Leibniz Institute for Solid State and Materials Research, 01069 Dresden, Germany; 9 Institute of Functional Nano & Soft Materials (FUNSOM), Soochow University, Suzhou, Jiangsu 215123 China; 10 Physikalische und Theoretische Chemie, Universität Siegen, 57068 Siegen, Germany

## Abstract

Unsupported non-bridged uranium–carbon double bonds have long been sought after in actinide chemistry as fundamental synthetic targets in the study of actinide-ligand multiple bonding. Here we report that, utilizing *I*_h_(7)-C_80_ fullerenes as nanocontainers, a diuranium carbide cluster, U=C=U, has been encapsulated and stabilized in the form of UCU@*I*_h_(7)-C_80_. This endohedral fullerene was prepared utilizing the Krätschmer–Huffman arc discharge method, and was then co-crystallized with nickel(II) octaethylporphyrin (Ni^II^-OEP) to produce UCU@*I*_h_(7)-C_80_·[Ni^II^-OEP] as single crystals. X-ray diffraction analysis reveals a cage-stabilized, carbide-bridged, bent UCU cluster with unexpectedly short uranium–carbon distances (2.03 Å) indicative of covalent U=C double-bond character. The quantum-chemical results suggest that both U atoms in the UCU unit have formal oxidation state of +5. The structural features of UCU@*I*_h_(7)-C_80_ and the covalent nature of the U(f^1^)=C double bonds were further affirmed through various spectroscopic and theoretical analyses.

## Abstract (web summary)

While metal–carbon double bonds are common in transition metal chemistry and catalysis, unsupported uranium–carbon double bonds remain highly challenging to prepare. Here, the authors stabilize and characterize a U=C=U cluster containing unusually short, unsupported double bonds inside an *I*_h_(7)-C_80_ fullerene cage.

## Introduction

Understanding the nature of actinide-ligand multiple bonding remains a modern day challenge owing to the complex electronic structures of these elements, and as a consequence, our comprehension of their chemistries lags behind that of the more commonly studied transition metals^1–5^. Importantly, recent developments in the synthesis and study of molecular uranium complexes containing a variety of U=E(L) or U≡E(L) moieties (E = O, S, Se, Te, N, NR, P, As; L = other ligands) have provided valuable insights regarding the participation of the 5f and 6d valence orbitals in chemical bonding^1,2,6–27^. Conspicuously missing from this list are U=C bonds, specifically those which lack ancillary heteroatom or chelating support. While metal–carbon double bonds (i.e., Schrock carbenes) are common in transition metal chemistry and catalysis, unsupported U=C bonds have long remained a major and outstanding synthetic target. Some success towards the synthesis of An=C bonds has been realized through the use of heteroatom stabilizing chelating ligands such as U=[C(Ph_2_PNSiMe_3_)_2_](O)Cl_2_ or U=[C(Ph_2_PS)] (BH_4_)_2_ (THF)_2_, among others^4,27–33^. In a few rare instances, the isolation of non-chelated U=C bonds has been achieved in complexes such as (*η*^5^-C_5_H_5_)_3_U=CHPMe_2_Ph and [N(SiMe_3_)_2_]_3_U=CHPPh_3_, using formally dianionic ylidic ligands^31,34^. Electronically, the U=C units found in these methanediide ([κ^3^-CL_2_]^2−^) or ylidic ([C(H)(PR_3_)]^2−^) examples are best described as highly polarized, nucleophilic carbenes with σ and modest π bonding overlap between uranium and carbon. The α-carbon bonded heteroatom(s) (e.g., phosphorous) aids the delocalization of the carbon-centered charge accumulation. While compounds possessing unsupported U=C bonds are difficult to prepare under typical synthetic conditions, Andrews and co-workers have identified alkylidene and even alkylidyne species such as H_2_C=UX_2_ and HC≡UX_3_ (X= H, F, Cl or Br) in low-temperature noble-gas matrix isolation experiments, providing evidence for such bonding motifs^35–37^. However, these compounds are too reactive to be isolated under typical synthetic conditions. The knowledge gained from studying stable compounds featuring U=C multiple-bonds can be extended to uranium carbide ceramics, which offer improved thermal density and higher conductivity over current UO_2_ nuclear fuels^38–40^.

Based on our recent success with the isolation of new actinide endohedral fullerenes, we turned our attention towards the synthesis and isolation of carbon cages encapsulating clusters that possess U=C bonds. The internal hollow cavity of C_2*n*_-fullerenes can encapsulate and stabilize novel metallic clusters, especially some which are highly reactive and virtually impossible to prepare independently^41–43^. Recently, we reported the first crystallographically characterized examples of actinide endohedral metallofullerenes (Th@C_82_ and U@C_2*n*_, 2*n* = 74, 82) and described their unique electronic properties^43,44^. We therefore hypothesized that fullerene cages would provide an ideal architecture and electronic environment to trap and stabilize unique actinide clusters with novel bonding motifs.

Herein, using the *I*_h_(7)-C_80_ fullerene cage as a molecular nanocontainer, we report, to the best of our knowledge, the first structurally characterized example of unsupported uranium U=C bonds found in UCU@*I*_h_(7)-C_80_, possessing the unprecedented dimetallic-carbide cluster U=C=U. X-ray crystallographic analysis reveals two very short U=C bonds of 2.033(5)/2.028(5) Å, with an unexpected nonlinear U=C=U bond angle of 142.8(3)°.

## Results

### Synthesis of U_2_C@C_80_

U_2_C@C_80_ was synthesized by the Krätschmer–Huffman arc discharge method^45^. Graphite rods, packed with graphite and finely dispersed U_3_O_8_, were vaporized in an arcing chamber under a He atmosphere. The compound was isolated and purified using a multistage high-performance liquid-chromatography protocol (HPLC). The composition and purity of the isolated U_2_C@C_80_ was confirmed by high-resolution matrix-assisted laser desorption-ionization time-of-flight positive-ion-mode mass spectrometry (MALDI-TOF/MS), which presents a prominent molecular ion peak with a mass-to-charge ratio of *m*/*z* = 1448.103 (Supplementary Fig. 1), corresponding to the [U_2_C_81_]^+^ empirical formula. The mass spectral isotopic distribution pattern matches the theoretically predicted one, thus confirming the molecular composition. In addition, an energy dispersive spectroscopic analysis of the purified sample was employed to determine the elementary composition of the compound. The spectrum shows characteristic peaks of uranium and carbon (Supplementary Fig. 2), which confirmed the assignment of this molecule to U_2_C_81_. Moreover, the powder X-ray diffraction pattern of U_2_C@C_80_ proves that the purified sample of U_2_C@C_80_ used for the experimental characterizations below, exists as a pure phase (Supplementary Fig. 5).

### Molecular structure of UCU@*I*_h_(7)-C_80_·[Ni^II^-OEP]

**Fig. 1:**
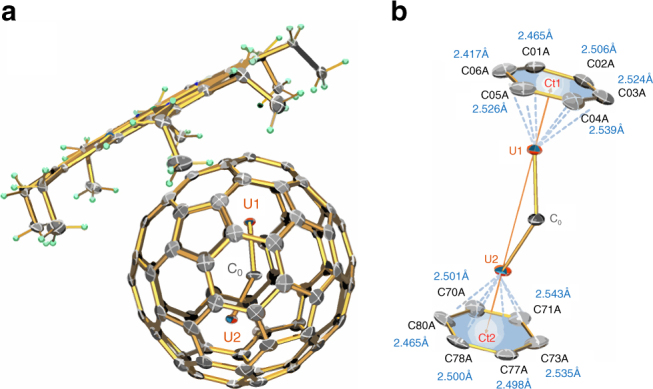
ORTEP drawing of UCU@*I*_h_(7)-C_80_·[Ni^II^-OEP] with 40% probability ellipsoids. **a** UCU@*I*_h_(7)-C_80_·[Ni^II^-OEP] structure showing the relationship between the fullerene cage and the [Ni^II^-OEP] ligands. The two U1/U2 sites have common occupancy of 0.853(3). Four minor U sites (Supplementary Fig. 6) and the solvent molecules are omitted here for clarity. **b** Fragment view showing the interaction of the major U1–C_0_–U2 cluster with the closest aromatic ring fragments of the cage with centers Ct1 and Ct2. The orange line connects Ct1–U1–U2–Ct2

The molecular structure of UCU@C_80_ was determined by single-crystal X-ray diffraction analysis. Slow diffusion of nickel(II) octaethylporphyrin (Ni^II^-OEP) in benzene into a CS_2_ solution of UCU@C_80_ yielded black UCU@*I*_h_(7)-C_80_·[Ni^II^-OEP] cocrystals (**1**). In cocrystal **1**, UCU@C_80_ is observed to adopt a slightly distorted icosahedral *I*_h_(7)*-*C_80_ cage structure (Fig. 1a) that is highly ordered. Similarly, inside the fullerene cage, the central carbon atom C_0_ of the endohedral UC_0_U unit is fully ordered, while the U atoms are slightly disordered. Two major U positions (U1 and U2) have common dominant occupancy of 0.853(3), while the residual occupancies are located on both sides within ca. ½ to 1 Å distance from the two main positions, possibly indicating some large amplitude motions of the UC_0_U cluster (Supplementary Fig. 6). The distances between the major U1,2 sites and the carbons of the adjacent aromatic rings of the cage all lie within a narrow range of around 2.50 Å (2.471(5) to 2.543(5) Å, Fig. 1b), corresponding to distances of the ring centers Cti to Ui of 2.042(1) Å and angles Cti−Ui−C_0_≈159(1)°. The U–C_0_ bonds are 2.03 Å (U1–C_0_ = 2.033(5) Å, U2–C_0_ = 2.028(5) Å), forming a nearly linear Ct1–U1—U2–Ct2 chain (Fig. 1b) bridged by a non-linear –C_0_– carbide anion.

**Fig. 2:**
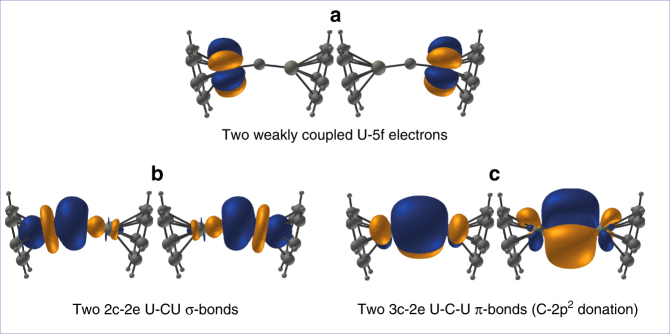
Natural Localized Molecular Orbitals (NLMOs) of sandwiched UCU. The UCU@(C_7_H_7_)_2_ molecule formally consists of a (UCU)^6+^ unit between two aromatic (C_7_H_7_)^3−^ rings. Spin–orbit coupled multi-reference all-electron relativistic wavefunction calculations (state-averaged CASSCF) were analyzed with the NBO program [S27]. The contour values of the NLMOs generated thereby are ±0.042√(e/Å^3^). **a** The two 1-center U-5f^1^-type NLMOs, each occupied by one electron. **b** The U–C (left) and C–U(right) 2-center 2-electron (2c2e) σ-pair NLMOs, each consisting of 67% C-2sp^1^, 31% U-5f^½^ 6d and 2% tail-contribution from the other U. **c** The vertical π-type and the in-plane dominantly π-type U–C–U 3-center 2-electron (3c2e) NLMOs of 65% C-2p_s_ and 17% of each of the two U-5f^½^ 6d

For hexa-coordinated uranium, Pyykkö found that two short and strong axial and four long and weak equatorial bonds are energetically near-equivalent to two long and weak axial and four short and strong equatorial bonds^46,47^. Therefore, particularly short axial bonds are expected when additional equatorial coordination is absent, as in the present case. Indeed, most U-Ct(arene) distances are reported between 2.5 and 2.9 Å, for instance 2.651(4) to 2.698(4) Å for the dinuclear U(V) inverse sandwich complex [{U^V^(Ts)}_2_(η^6^:η^6^-C_6_H_5_Me)]^48^. However, by far the most salient features of **1** are the unusually short U–C_0_ bond lengths, (U1–C_0_ = 2.033(5) Å, U2–C_0_ = 2.028(5) Å) and the unusual bond angle of 142.8(3)° for U1–C_0_–U2 (Table 1). In fact, the shortest U–C bond distance in poly-coordinated U-complexes to date is 2.184(3) Å, reported by Liddle et al. for the U(VI) carbene complex U=[C(Ph_2_PNSiMe_3_)_2_](O)Cl_2_^27^. Interestingly, the U–C bond lengths of the UC_0_U unit are between the 2.067(7) Å and 1.948(7) Å, theoretically calculated distances for uranium alkylidene and alkylidyne complexes U=CH_2_(H)F and U≡CH(Cl)_3_^35–37^, and are nearly identical to the 2.01 Å distance calculated from the sum of the covalent radii^49^. Furthermore, the U–C_0_ distances in **1** are longer than the triple bond of the U≡O unit (1.78 Å) in the uranyl dication UO_2_^2+^, the U≡NR bonds (1.844(6) Å) of U(N^t^Bu)_2_(I)_2_(THF)_2_), and the terminal U≡N bond (1.83(2) Å for the uranium nitride complex (Tren^TIPS^)UN), even when accounting for differences in the atomic radii of O, N, and C of ca. 0.05 Å each^18,23,50^.

**Table 1:** Calculated and experimentally derived geometric parameters of UCU groups

Geometric parameter	Calc.^a^ UCU@(3I)_2_ (molecule)	Calc.^a^ UCU@(C_7_H_7_)_2_ (molecule)	Calc.^a^ UCU@*I*_h_(7)-C_80_ (molecule)	Exptl.^b^ UCU@*I*_h_(7)-C_80_ •[Ni^II^-OEP] (crystal)
U–C /Å	2.024	2.074	2.022	2.033/2.028
U∙∙∙U /Å	3.918	4.118	3.917	3.849
Ligand→U /Å	2.33^c^	2.52–2.55	2.47–2.51	2.47–2.54
U–C–U / ^o^	150.9	166.1	151.3	142.8
Fullerene C–C /Å	—	—	100 × 1.43 ± 0.01	110 × 1.43 ± 0.03
‘π-Donating’ C–C /Å	—	14 × 1.43 ± 0.01	20 × 1.46 ± 0.01	10 × 1.48 ± 0.01

^a^From scalar relativistic ZORA Kohn–Sham PBE VTZp approaches

^b^From crystal structure analysis by X-ray diffraction

^c^For comparison of I−U with C−U, we have subtracted the I–C bond-radii difference of 0.55 Å from the actual I–U distance

Altogether, the bond lengths observed for **1** strongly support an Arene≡U=C=U≡Arene structure possessing two uranium–carbon double bonds formed by a bridging carbide atom, formally C^4−^. The bending of the U=C=U cluster of **1** was an unexpected exception to the common linear geometries of *sp*_*σ*_^1^–p_π_^2^ hybridized carbon atoms found for main-group molecules of type E=C=E (E=O, NR, CR_2_), though a few cases with angles between 180° and 90° have been theoretically proposed and observed in recent years^51^.

### Computational studies of molecular structure and bonding in UCU@*I*_h_(7)-C_80_

Quantum-chemical calculations were performed to further verify the unique structural parameters of UCU@*I*_h_(7)-C_80_ and gain insight about the bonding of the encapsulated bent U=C=U cluster. The molecular structure was modeled for free UCU@*I*_h_(7)-C_80_ and for LUCUL (L = C_7_H_7_, 3I) model molecules, using quasi-relativistic density-functional (DF) approximations (Supplementary Figs. 13, 14) and ab-initio CASSCF(10e,12o)-PT2 approaches (Table 1). Additional information of the structure, orbitals and electronic states of LUCUL is available in Supplementary Figs. 15–19 and Supplementary Tables 3–5. For the three calculated cases, the formal oxidation state^52^ is identified as 5+ for each U, derived from the formal [L^3−^≡>U^5+^<=C^4−^=>U^5+^<≡L^3−^] Lewis structure, where L^3−^≡>stands for the cage arene units that accept 3 electrons from each uranium atom and forms 3 dative pair bonds with the nearly empty U-5f6d valence shell. In the case of UCU@*I*_h_(7)-C_80_, the two L^3−^ units are the ligating arene units of the encapsulating C_80_^6–^ closed-shell cage. The C and I atoms of the model units (C_7_H_7_) and (3I) have similar electronegativity as C of (C_80_), and the model units also become closed-shell ligands upon accepting 3 electrons from each U. The effective physical partial charges in all three cases remain small, around −1 for formal C^4−^, around +½ to +1 for formal U^5+^, and small for the ligand atoms of C_80_, 2C_7_H_7_ or 6I (Supplementary Table 1).

The atomic free valence and the spin and orbital populations (Supplementary Table 1) indicate a single f-electron on each U atom. The experimental U–C_0_ bond lengths, though unprecedentedly short (2.03 Å), are well reproduced in all three computed models (2.02 Å for ligands C_80_ or (I_3_)_2_, 2.07 Å for (C_7_H_7_)_2_). Further, the observed unusual U–C_0_–U bond angle of 142.8° is comparable to those computed for UCU@C_80_ (151.3°) and (3I)UCU(3I) (150.9°), while the bulkier ligands of (C_7_H_7_)UCU(C_7_H_7_) led to 166.1°, which is still far from linear (Table 1). Neither the bond lengths nor the bond angle of the UCU unit appear to be significantly distorted by the encapsulation in C_80_.

**Fig. 3:**
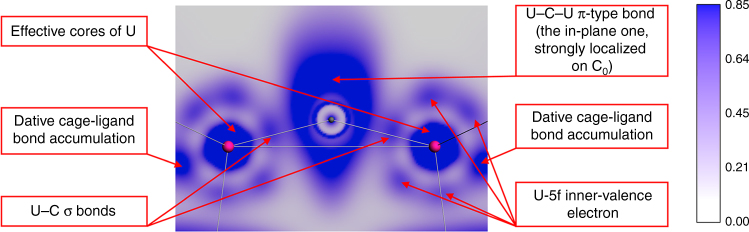
ELF-plot of ligated UCU in the molecular plane. The left and right ligands are two (I_3_) groups. ELF is the ‘electron localization function’ of Becke and Edgecombe, showing electronic accumulations in the inner atomic shells, in the atomic lone-pair regions, and in covalent and dative bond regions

We chose the localized equivalent molecular orbital picture of the two simpler model systems to sketch the correlated and spin–orbit coupled valence electronic structure of ligated UC_0_U. (C_7_H_7_)UCU(C_7_H_7_) (Fig. 2) and (3I)UCU(3I) (Supplementary Fig. 15) gave very similar results. Each U forms a strong σ-bond to the central C_0_ atom through covalent overlap of hybridized U(5 f6d) orbitals with C(2s2p) hybrids. The U–C bonds of dominant π-character can be described as three-center two-electron (3c2e) bonds that possess some σ-admixture. The large electronegativity difference of C and U (1.3 Pauling units) leads to bond-pair polarization toward C_0_, especially for the π-type pairs, which are strongly localized on C_0_ and lead to its effective negative charge. The U–C interactions are characterized by Mayer bond orders (BO) of 1.4 from DF calculations (comparable to 1.6 for the U=As double bond in [K(B15C5)_2_] {[N(CH_2_CH_2_NSi^*i*^Pr_3_)_3_] U=AsH})^26^ and by a Roos effective bond order (EBO) of 1.9 (counting the formally bonding—antibonding NOs from our CASSCF calculation of (C_7_H_7_)UCU(C_7_H_7_), which substantiates the U=C double bond character).

The bonding motif is nicely illustrated by the electron localization function (ELF) map in Fig. 3, which shows the electron–density accumulation of the two two-center U–C σ-bonds and of the in-plane three-center U–C–U π-type bond, as well as the multiple maxima of the inner-valence non-bonding 5f electrons. Thus, the bending of the U=C=U unit can be interpreted in terms of the localized negative charge build-up on C_0_, which generates lone electron-pair density and gives rise to an sp^1^/sp^2^-hybridized type geometry at carbon with bond angles in the middle between 120° and 180°. This charge accumulation is partially offset by donation into the U(5f6d) orbitals. Thus the bonding model can be described as highly polarized U=C interactions with partial π-overlap strengthened by electrostatic attractive forces between the cationic U^5+^ atoms and the anionic C_0_^4−^ carbide bridge.

### Spectroscopic properties of UCU@*I*_h_(7)-C_80_

Nuclear Magnetic Resonance (NMR) spectroscopy was used to characterize UCU@*I*_h_(7)-C_80_. The ^13^C NMR spectrum of UCU@*I*_h_(7)–C_80_ measured at 298 K shows only two sharp signals at 138.53 and 125.14 ppm with a 3:1 intensity ratio, corresponding to the sets of 60 and 20 equivalent carbon atoms of the unperturbed *I*_h_–C_80_ fullerene cage (Supplementary Fig. 7). The apparent symmetry is likely due to fast and large-amplitude librations of the cage, similar to those proposed for M_2_@*I*_h_-C_80_ (M = La, Ce) and M_3_N@C_80_ (M = Sc, Y)^42,53,54^. Unfortunately, no signal was detected for the single bridging C_0_ carbon atom, likely due to limited sample amount, as well as to paramagnetic broadening effects from the unpaired 5f^1^ electrons on each uranium. Noteworthy is that the ^13^C NMR spectrum measured at lower temperature (i.e., 283 K) shows slightly shifted signals at 138.35 and 124.65 ppm, respectively, with a slightly larger chemical shift difference (Δ*δ* = 13.70 ppm) than those observed at 298 K (Δ*δ* = 13.39 ppm). Such a trend is very similar to that observed for Ce_2_@*I*_h_–C_80_^55^, consistent with the paramagnetic nature of UCU (see below) and with the presence of an unpaired *f* electron on the formal U^5+^ (5f^1^6d^0^7s^0^) ions (Supplementary Table 1)^54^. On the other hand, the slight temperature dependence of Δ*δ*/Δ*T* = 0.02 ppm/K might also be accounted for by the libration of the UCU cluster that neutralizes the paramagnetic effects (mainly pseudocontact interactions) for all carbon atoms.

**Fig. 4:**
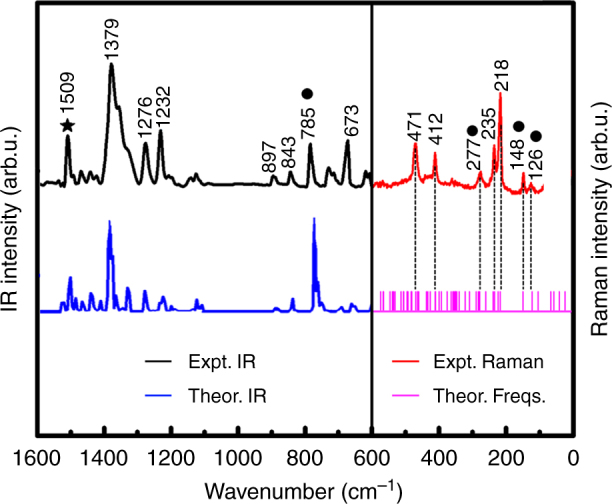
Observed and theoretically predicted vibrational features of UCU@*I*_h_(7)-C_80_. The left upper curve (black) presents the observed Infrared absorption (IR) spectrum vs. wavenumber from 1600 to 600 cm^‒1^, with quantum-chemical density-functional simulation below (in blue). On the right is the observed Raman emission (in red) vs. wavenumber from 600 to 100 cm^‒1^, with calculated wavenumbers (‘Theor. Freqs.’) in the 600–0 cm^‒1^ range (below in magenta). Observed local UCU vibrations above 100 cm^‒1^ are indicated by heavy dots. Observed and calculated spectral C_80_-cage features represent either a single mode, or an overlay of several near-degenerate ones. indicates a contamination by CS_2_

We also utilized Fourier transform infrared absorption (FTIR), Raman emission and photoluminescence (PL) spectroscopies together with quantum-chemical calculations to further characterize UCU@*I*_h_(7)-C_80_. An experimental and theoretical spectral overview is presented in Fig. 4. The high-wavenumber range from 1600 to 1100 cm^‒1^ contains 104 carbon cage C–C stretching vibrations, resembling those of other *I*_h_-C_80_ based endohedral fullerenes such as Ln_3_N@C_80_^41,56^. Typical characteristics are the major overlaid bands around 1380 cm^−1^ and the featureless gap between 1100 and 900 cm^‒1^. The remaining 130 collective deformations originating from bending and torsional motions of the carbon cage atoms show up between 900 and 200 cm^−1^.

Quantum-chemical calculations suggest that an endohedral cluster of *n* atoms (here *n* = 3) shows 3 frustrated translational rocking modes against the cage (at 33, 59, and 115 cm^‒1^), 3 frustrated torsional wagging modes (at 16, 50, and 142 cm^‒1^), and 3*n* – 6 = 3 internal vibrations. The latter comprise the UC_0_U bending mode (at 97 cm^‒1^), the symmetric stretch (coupled to near-degenerate cage deformations from 250 to 300 cm^‒1^), and the asymmetric stretch (at 780 cm^‒1^). The observed weak features in the Raman spectrum above 100 cm^‒1^ at 126, 148, and 277 cm^‒1^ agree reasonably well with the predicted UCU modes given above (in bold italics, estimated as weakly Raman active). Notably, the asymmetric UCU stretch appears as a pronounced feature in the IR spectrum at 785 cm^‒1^, which is consistent with the high negative charge on the C_0_ atom of the endohedral UCU cluster, and with the crystallographic observation of very short axial U=C bonds.

Further, an experimental violet-blue photo-luminescence progression of U_2_C@*I*_h_(7)-C_80_ starting at a wavelength of 430 nm (~2.95 eV) in steps of ca. 1425 cm^‒1^ (Supplementary Fig. 8) may originate from a ligand-to-metal charge-transfer excitation of the highest occupied molecular orbital, which has dominant density on the fullerene cage near the U atoms. There are also C_ring_-breathing vibrations around 1400 cm^−1^ localized in the spatial C_ring_–U–C–U–C_ring_ region.

The redox properties of UCU@*I*_h_(7)-C_80_, as investigated by means of cyclic voltammetry, show a surprisingly small electrochemical gap of only 0.83 eV (Table 2, Supplementary Fig. 9). Most reported transition metal cluster fullerenes of *I*_h_(7)-C_80_ have gaps larger than 1 eV^41^. Typical M_3_N@*I*_h_(7)-C_80_ fullerenes have gaps of 1.8–2.2 V. In particular, the first reduction potential of UCU@*I*_h_(7)-C_80_ (−0.41 V) is much more positive than that of any other reported M_3_N@*I*_h_(7)-C_80_ fullerene^57^, e.g., −1.26 V for Sc_3_N@*I*_h_(7)-C_80_^58^ and −0.94 V for TiLu_2_C@*I*_h_(7)-C_80_^59^. UCU@*I*_h_(7)-C_80_ obviously exhibits a much better electron-accepting ability than other *I*_*h*_(7)-C_80_ clusterfullerenes. This significant difference between UCU@*I*_h_(7)-C_80_ and other reported M_3_N@C_80_ fullerenes indicates a major difference of their electronic structures, which may be caused by the encapsulation of the U=C0=U cluster with an open d–f shell electronically interacting with the cage. Interestingly, when compared to most other *I*_h_(7)-C_80_ cluster fullerenes, the redox potentials of UCU@*I*_h_(7)-C_80_ are close to those of dilanthanide-fullerene, such as La_2_@*I*_h_(7)-C_80_, which exhibits an electrochemical gap of 0.87 eV^60^. In general, the electrochemical gaps are related to the HOMO-LUMO gaps of the closed-shell fullerene subsystems. However, in the present case, the encapsulated U=C_0_=U cluster has a low-lying open U-5f shell, causing an abnormally small f–f gap of the UCU@*I*_h_(7)-C_80_ molecule. The reversible reduction of UCU@*I*_h_(7)-C_80_ is also quite remarkable, since M_3_N@*I*_h_(7)-C_80_ fullerenes with group 3 metals exhibit irreversible reductions^57^.

**Table 2:** Redox potentials and electrochemical gaps of UCU@*I*_h_(7)–C_80_, La_2_@*I*_h_(7)–C_80_, and Sc_3_N@*I*_h_(7)–C_80_

Fullerenes	*E* ^2+/+ a^	*E* ^*+/*0 a^	*E* ^0/− a^	*E* ^-/2− a^	*E* ^2−/3− a^	*E*_gap_^b^	Ref.
UCU@*I*_h_(7)-C_80_	+1.05^d^	+0.42^c^	−0.41^c^	−1.34^c^		0.83	This work
La_2_@*I*_h_(7)-C_80_	+0.95^c^	+0.56^c^	−0.31^c^	−1.72^c^	−2.13^d^	0.87	^60^
Sc_3_N@*I*_h_(7)-C_80_		+0.97^c^	−1.26^d^	−1.62^d^	−1.82^d^	2.23	^58^

^a^Redox potentials in V vs. ferrocene couple

^b^Electrochemical gaps in eV

^c^Half-wave potential (reversible redox process)

^d^Peak potential (irreversible redox process)

The XPS spectrum of UCU@*I*_h_(7)-C_80_ was also recorded (Supplementary Fig. 10). The U-4f_7/2_ ionization appears at 378.6 eV. In general, ionization energies increase with the formal oxidation state, e.g., from the U-4f_7/2_ value of ca. 377_1/3_ eV for the neutral uranium metal to ca. 381_3/4_ eV for some uranyl(VI) salts. The present value of 378.6 eV is consistent with the very low effective charges on U as computed for UC_0_U@C_80_ (see below, U ~ +1.0e; C_o_ ~ −1.0e, C_fullerene_ ~ ± 0.0e), caused by the strong electron pair donation of formal C_80_^6−^ and C_0_^4−^ to the formal U^5+^ ions.

To gain further insight into the unique electronic characteristics of the U=C=U cluster in UCU@C_80_, the magnetic susceptibility was measured (see Supplementary Fig.11 and Methods). The magnetic curves exhibit two regimes, (i) one at “high temperatures” from 300 K down to ca. 60 K, and (ii) another one at “low temperatures” from ca. 40 K down to 2 K. The “high temperature” region exhibits a huge, basically temperature-independent paramagnetism (TIP) of yet unknown origin. It is unknown for uranium complexes with common ligands^21,22,48,61–65^ and may possibly be due to the cooperative coupling of UCU and C_80_ in the solid and related to the low band-gap calculated for UCU@C_80_ (see Supplementary “Quantum Computational Methods”).

At “low temperatures”, we observed a Curie type paramagnetism ~μ^2^/T on top of the TIP, with net μ-values increasing with temperature from below to above a μ_Bohr_, which appears compatible with hardly coupled unique units of type η^3^L≡U(V)=C=U(V)≡Lη^3^, i.e., the endohedral di-uranium carbide fullerene molecules. The “low-temperature” literature values of μ_eff_^21,22,48,61–65^ for mono- and bridged di-metallic U(V) and U(IV) complexes, typically increase, too, from low values up to 3 μ_Bohr_. It shows the expected temperature dependent weak coupling of two spins and with the two partially quenched U-f±3 orbital angular momenta, the experimental curves being reasonably reproduced by numerical simulations (see Supplementary Method “Quasi-relativistic correlated ab initio approaches” on the (C_7_H_7_)UCU(C_7_H_7_) model).

Attempts to further resolve the electronic structure using EPR spectroscopy were unsuccessful, as no clearly defined signal was observed at 4 K (Supplementary Fig. 12). Factors such as near degenerate electronic states and cage shielding effects as well as the huge TIP complicate the analysis. Thus, additional studies of the electronic properties of this unique and unprecedented system are warranted and will be communicated in due time.

## Discussion

In summary, the overall agreement between the crystallographic, luminescence, Raman, IR, core-electronic, magnetic and voltammetric results, and the quantum-computational findings, conclusively show that a pure UCU@*I*_h_(7)-C_80_ compound was synthesized, which consists of a weakly perturbed *I*_h_-C_80_ cage and a sandwiched, bent and strongly bound polar endohedral near-symmetric U=C=U unit. The quantum-chemical results suggest that both U atoms in the UCU unit have a formal +5 oxidation state. The encapsulation causes little bond length or angle deformation of the UCU fragment. The encapsulation protects the axially ligated U atoms from binding with harder or more electronegative ligands like O or N (as compared to carbon in the forms of C_0_^4−^ and C_80_^6−^) so that C_0_ can participate in primarily axial L≡>U=C_0_ bonding at unusually short U–C_0_ distances of 2.03 Å, and is not pushed into the weaker bonding equatorial plane of an E=U=E building block. The discovery of unsupported U=C bonding in a molecular compound confirms the distinction between “diagonal” and “poly-coordinated” uranium, and between “axial” covalent and “equatorial” dative bonding mechanisms. The work reported here offers a deeper understanding of the fundamentals of uranium bonding properties. This study also demonstrates that fullerene cages can be utilized as effective nanocontainers to stabilize and study rare and reactive clusters which contain actinide metal–ligand bonds.

## Methods

### Synthesis, separation and purification of U_2_C@*I*_h_(7)-C_80_

A soot containing uranium fullerenes was synthesized by a direct-current arc discharge method. Graphite rods of U_3_O_8_ and graphite powder were annealed in a tube furnace at 1000 °C for 20 h under an Ar atmosphere and then burned in the arcing chamber under a 300 Torr He atmosphere. In total 1.87 g of graphite powder and 1.83 g of U_3_O_8_ (24:1 atom number ratio) were packed in each rod (6.7 g, without filling). The collected raw soot was refluxed in chlorobenzene under Ar atmosphere for 12 h and the solution was filtered. After solvent removal, the fullerenes were extracted by dissolving in toluene. On average ca. 140 mg of crude fullerene mixture per rod was obtained. In total, 60 carbon rods were burned in this work. Separation and purification of U_2_C@*I*_h_(7)-C_80_ was achieved by a three-stage HPLC procedure (Supplementary Fig. 3, using columns including a Buckyprep M column (25 × 250 mm^2^, Cosmosil, Nacalai Tesque, Japan), a Buckprep D column (10 × 250 mm^2^, Cosmosil, Nacalai Tesque, Japan) and a Buckprep column (10 × 250 mm^2^, Cosmosil, Nacalai Tesque, Japan). Toluene was used as the mobile phase and the UV detector was adjusted to 310 nm for fullerene detection. The HPLC procedures and corresponding MALDI-TOF spectra for the isolated fractions are shown in Supplementary Figs. 3, 4. In total, ca. 2 mg of highly purified U_2_C@C_80_ was obtained for characterization.

### Single crystal and powder X-ray diffraction analysis

Black co-crystals of UCU@*I*_h_-C_80_·[Ni^II^-OEP] were obtained by allowing the benzene solution of [Ni^II^-OEP](1.2 mg/mL, 0.8 mL) and the CS_2_ solution of UCU@*I*_h_-C_80_(1 mg/mL, 0.5 mL) to diffuse together. X-ray diffraction data were collected at 120 K using a diffractometer (APEX II; Bruker Analytik GmbH) equipped with a CCD collector. The Multiscan method was used for the absorption correction. The structure was resolved using direct methods (SIR2004) and refined on *F*^*2*^ with the full-matrix least-squares approach using SHELXL2014^66,67^ within the WinGX package^68^.

Crystal Data for UCU@*I*_h_-C_80_·[Ni^II^OEP]·1.5C_6_H_6_·CS_2_: C_127_H_53_N_4_NiS_2_U_2_, *M* = 2159.70, 0.05 × 0.05 × 0.03 mm^3^, monoclinic, P *21/c* (No. 14), *a* = 17.678(4) Å, *b* = 16.970(3) Å, *c* = 26.695(5) Å, *β* = 106.65(3)^°^, *V* = 7673(3) Å^3^, *Z* = 4, *ρ*_*calcd*_ = 1.934 g cm^−3^, *μ* = 4.578 mm^−1^, *θ* = 2.688–27.092°, *T* = 120 K, *R*_1_ = 0.0610, *wR*_2_ = 0.1045 for all data; *R*_1_ = 0.0388, *wR*_2_ = 0.0610 for 16,771 reflections (*I* > 2.0σ(*I*)) with 1278 parameters and 784 restraints. Goodness of fit indicator 1.007. Maximum residual electron density 2.096 e Å^−3^. For further details see the Supplementary Data 1, 2.

In addition, a powder X-ray diffraction analysis of UCU@C_80_ was conducted at the Shanghai Synchrotron Radiation Facility (SSRF) with wavelength *λ* = 0.6199 Å, and the intensity vs. 2*θ* pattern was recorded, see Supplementary Fig. 5.

### Computational methods

Open-shell Kohn–Sham single-reference calculations were carried out with the ADF-2016, Gaussian-09 and ORCA-4.0 software, applying scalar and spin–orbit-coupled quasi-relativistic approaches (RECP or ZORA) with triple-ζ double-polarized basis sets. Bonding analyses were then performed using the NBO5.0 and Multiwfn codes. Multi-reference CASSCF, CASPT2, and RAS-SI/SO wave-function calculations were performed using the MOLCAS-V8.1 software applying the quasi-relativistic DKH Hamiltonian. Further details are given in the Supplementary “Quantum Computational Methods”.

### Data availability

The coordinates for the X-ray structure of **1** are available free of charge from the Cambridge Crystallographic Data Centre under deposition no. CCDC 1572891. All other data supporting the findings of this study are available from the corresponding authors on request.

## Electronic supplementary material

Supplementary Information

Peer Review File

Description of Additional Supplementary Files

Supplementary Data 1

Supplementary Data 2

## References

[1] Hayton TW (2010). Metal-ligand multiple bonding in uranium: structure and reactivity. Dalton Trans..

[2] Hayton TW (2013). Recent developments in actinide-ligand multiple bonding. Chem. Commun..

[3] Liddle ST (2015). The renaissance of non-aqueous uranium chemistry. Angew. Chem. Int. Ed..

[4] Gregson M, Wooles AJ, Cooper OJ, Liddle ST (2015). Covalent uranium carbene chemistry. Inorg. Chem..

[5] Patel D, Liddle ST (2012). f-Element-metal bond chemistry. Rev. Inorg. Chem..

[6] Fortier S, Brown JL, Kaltsoyannis N, Wu G, Hayton TW (2012). Synthesis, molecular and electronic structure of U^V^(O)[N(SiMe_3_)_2_]_3_. Inorg. Chem..

[7] Lewis AJ, Mullane KC, Nakamaru-Ogiso E, Carroll PJ, Schelter EJ (2014). The inverse trans influence in a family of pentavalent uranium complexes. Inorg. Chem..

[8] Lewis AJ, Carroll PJ, Schelter EJ (2013). Stable uranium(VI) methyl and acetylide complexes and the elucidation of an inverse trans influence ligand series. J. Am. Chem. Soc..

[9] Kosog B, La Pierre HS, Heinemann FW, Liddle ST, Meyer K (2012). Synthesis of uranium(VI) terminal oxo complexes: molecular geometry driven by the inverse trans-influence. J. Am. Chem. Soc..

[10] Fortier S, Wu G, Hayton TW (2010). Synthesis of a nitrido-substituted analogue of the uranyl ion, [N═U═O]_+_. J. Am. Chem. Soc..

[11] Hayton TW, Boncella JM, Scott BL, Batista ER (2006). Exchange of an imido ligand in bis(imido) complexes of uranium. J. Am. Chem. Soc..

[12] Brown JL, Fortier S, Lewis RA, Wu G, Hayton TW (2012). A complete family of terminal uranium chalcogenides, [U(E)(N{SiMe_3_}_2_)_3_]^−^ (E=O, S, Se, Te). J. Am. Chem. Soc..

[13] Brown JL, Fortier S, Wu G, Kaltsoyannis N, Hayton TW (2013). Synthesis and spectroscopic and computational characterization of the chalcogenido-substituted analogues of the uranyl ion, [OUE]^2+^ (E=S, Se). J. Am. Chem. Soc..

[14] Fortier S, Kaltsoyannis N, Wu G, Hayton TW (2011). Probing the reactivity and electronic structure of a uranium(V) terminal oxo complex. J. Am. Chem. Soc..

[15] Smiles DE, Wu G, Hayton TW (2014). Synthesis of uranium–ligand multiple bonds by cleavage of a trityl protecting group. J. Am. Chem. Soc..

[16] Smiles DE, Wu G, Hrobárik P, Hayton TW (2016). Use of ^77^Se and ^125^Te NMR spectroscopy to probe covalency of the actinide-chalcogen bonding in [Th(En){N(SiMe_3_)_2_}_3_^]−^ (E=Se, Te; n=1, 2) and their oxo-uranium(VI) congeners. J. Am. Chem. Soc..

[17] Rosenzweig MW (2016). Uranium(IV) terminal hydrosulfido and sulfido complexes: insights into the nature of the uranium-sulfur bond. Chem. Sci..

[18] Hayton TW (2005). Synthesis of imido analogs of the uranyl ion. Science.

[19] Andrez J (2016). Synthesis and reactivity of a terminal uranium(IV) sulfide supported by siloxide ligands. Chem. Sci..

[20] King DM, Liddle ST (2014). Progress in molecular uranium-nitride chemistry. Coord. Chem. Rev..

[21] Tsoureas N, Kilpatrick AFR, Inman CJ, Cloke FGN (2016). Steric control of redox events in organo-uranium chemistry: synthesis and characterisation of U(V) oxo and nitrido complexes. Chem. Sci..

[22] King DM (2012). Synthesis and structure of a terminal uranium nitride complex. Science.

[23] King DM (2013). Isolation and characterization of a uranium(VI)–nitride triple bond. Nat. Chem..

[24] Anderson NH (2017). Elucidating bonding preferences in tetrakis(imido)uranate(VI) dianions. Nat. Chem..

[25] Gardner BM (2014). Triamidoamine-uranium(IV)-stabilized terminal parent phosphide and phosphinidene complexes. Angew. Chem. Int. Ed..

[26] Gardner BM (2015). Triamidoamine uranium(IV)–arsenic complexes containing one-, two- and threefold U–As bonding interactions. Nat. Chem..

[27] Mills DP (2012). Synthesis of a uranium(VI)-carbene: reductive formation of uranyl(V)-methanides, oxidative preparation of a [R_2_C=U=O]^2+^ Analogue of the [O=U=O]^2+^ uranylion (R=Ph_2_PNSiMe_3_), and comparison of the nature of U^IV^=C, U^V^=C, and U^VI^=C double bonds. J. Am. Chem. Soc..

[28] Cooper OJ (2013). The nature of the UC double bond: pushing the stability of high-oxidation-state uranium carbenes to the limit. Chem. Eur. J..

[29] Lu EL (2014). Synthesis, characterization and reactivity of a uranium(VI) carbene imido oxo complex. Angew. Chem. Int. Ed..

[30] Arnold PL (2009). Organometallic actinides: Now U=C it. Nat. Chem..

[31] Cramer RE, Maynard RB, Paw JC, Gilje JW (1981). A uranium-carbon multiple bond. crystal and molecular structure of (η^5^-C_5_H_5_)_3_UCHP(CH_3_)_2_(C_6_H_5_). J. Am. Chem. Soc..

[32] Tourneux JC, Berthet JC, Mézailles N, Ephritikhine M (2011). Exploring the uranyl organometallic chemistry: from single to double uranium-carbon bonds. J. Am. Chem. Soc..

[33] Cantat T (2009). The U=C double bond: synthesis and study of uranium nucleophilic carbene complexes. J. Am. Chem. Soc..

[34] Fortier S, Walensky JR, Wu G, Hayton TW (2011). Synthesis of a phosphorano-stabilized U(IV)-carbene via one-electron oxidation of a U(III)-ylide adduct. J. Am. Chem. Soc..

[35] Lyon JT, Andrews L (2006). Formation and characterization of the uranium methylidene complexes CH_2_=UHX (X=F, Cl, and Br). Inorg. Chem..

[36] Lyon JT, Hu HS, Andrews L, Li J (2007). Formation of unprecedented actinide carbon triple bonds in uranium methylidyne molecules. Proc. Natl Acad. Sci. USA.

[37] Cho HG, Andrews L (2017). Matrix preparation and spectroscopic and theoretical investigation of small high oxidation-state complexes of groups 3-12, 14, lanthanide and actinide metal atoms: carbon-metal single, double and triple bonds. Coord. Chem. Rev..

[38] Du J, Jiang G (2017). A systematic theoretical study of UC_6_: structure, bonding nature, and spectroscopy. Inorg. Chem..

[39] Utton CA (2009). Laser melting of uranium carbides. J. Nucl. Mater..

[40] Frad, W. A. *Advances in Inorganic Chemistry and Radiochemistry* Vol. 11, 153–247 (Academic Press, New York and London, 1968).

[41] Popov AA, Yang S, Dunsch L (2013). Endohedral fullerenes. Chem. Rev..

[42] Stevenson S (1999). Small-bandgap endohedral metallofullerenes in high yield and purity. Nature.

[43] Wang Y (2017). Unique four-electron metal-to-cage charge transfer of Th to a C_82_ fullerene cage: complete structural characterization of Th@*C*_3v_(8)-C_82_. J. Am. Chem. Soc..

[44] Cai W (2017). Single crystal structures and theoretical calculations of uranium endohedral metallofullerenes (U@C_2n_, 2n = 74, 82) show cage isomer dependent oxidation states for U. Chem. Sci..

[45] Krätschmer W, Lamb LD, Fostiropoulos K, Huffman DR (1990). Solid C_60_: a new form of carbon. Nature.

[46] Pyykkö P, Zhao Y (1991). The large range of uranyl bond lengths: ab initio calculations on simple uranium-oxygen clusters. Inorg. Chem..

[47] Pyykkö P, Li J, Runeberg N (1994). Quasirelativistic pseudopotential study of species isoelectronic to uranyl and the equatorial coordination of uranyl. J. Phys. Chem..

[48] Patel D (2011). A formal high oxidation state inverse-sandwich diuranium complex: a new route to f-block-metal bonds. Angew. Chem. Int. Ed..

[49] Pyykkö P, Atsumi M (2009). Molecular double-bond covalent radii for elements Li–E112. Chem. Eur. J..

[50] Fortier S, Hayton TW (2010). Oxo ligand functionalization in the uranyl ion (UO_2_^2+^). Coord. Chem. Rev..

[51] Frenking G (2014). Dative bonds in main-group compounds: a case for more arrows!. Angew. Chem. Int. Ed..

[52] Karen P (2015). Oxidation state, a long-standing issue!. Angew. Chem. Int. Ed..

[53] Yamada M, Mizorogi N, Tsuchiya T, Akasaka T, Nagase S (2008). Metal atoms collinear with the spiro carbon of 6,6-open adducts, M_2_@C_80_(Ad) (M=La and Ce, Ad=adamantylidene). J. Am. Chem. Soc..

[54] Yamada M, Mizorogi N, Tsuchiya T, Akasaka T, Nagase S (2009). Synthesis and characterization of the *D*_5h_ isomer of the endohedral dimetallofullerene Ce_2_@C_80_: two-dimensional circulation of encapsulated metal atoms inside a fullerene Cage. Chem. Eur. J..

[55] Yamada M (2005). Positional control of encapsulated atoms inside a fullerene cage by exohedral addition. J. Am. Chem. Soc..

[56] Krause M (2001). Structure and sta.bility of endohedral fullerene Sc_3_N@C_80_: a Raman, infrared and theoretical analysis. J. Chem. Phys..

[57] Chaur ManuelN, Melin F, Ortiz, Angy L, Echegoyen L (2009). Chemical, electrochemical, and structural properties of endohedral metallofullerenes. Angew. Chem. Int. Ed..

[58] Zhang L, Chen N, Fan L, Wang C, Yang S (2007). Electrochemistry of Sc_3_N@C_78_ and Sc_3_N@C_80_ (*I*_h_): On achieving reversible redox waves of the trimetal nitride endohedral fullerenes. J. Electroanal. Chem..

[59] Svitova AL (2014). Endohedral fullerene with µ_3_-carbido ligand and titanium-carbon double bond stabilized inside a carbon cage. Nat. Commun..

[60] Suzuki T (1995). Electrochemistry and ab initio study of the dimetallofullerene La_2_@C_80_. Angew. Chem. Int. Ed..

[61] Rosen RK, Andersen RA, Edelstein NM (1990). [(MeC_5_H_4_)_3_U]_2_[μ-1,4-N_2_C_6_H_4_]: a bimetallic molecule with antiferromagnetic coupling between the uranium centers. J. Am. Chem. Soc..

[62] Spencer LP (2009). Cation–cation interactions, magnetic communication, and reactivity of the pentavalent uranium ion [U(N tBu)_2_]^+^. Angew. Chem. Int. Ed..

[63] Chen L (2016). A mixed-valent uranium phosphonate framework containing U^IV^, U^V^, and U^VI^. Chem. Eur. J..

[64] Schmidt AC, Heinemann FW, Lukens WW, Meyer K (2014). Molecular and electronic structure of dinuclear uranium Bis-μ-Oxo complexes with diamond core structural motifs. J. Am. Chem. Soc..

[65] Graves CR, Kiplinger JL (2009). Pentavalent uranium chemistry−synthetic pursuit of a rare oxidation state. Chem. Commun..

[66] Burla MC (2005). SIR2004: an improved tool for crystal structure determination and refinement. J. Appl. Crystallogr..

[67] Sheldrick G (2015). Crystal structure refinement with SHELXL. Acta Crystallogr. Sec. C.

[68] Farrugia L (2012). WinGX and ORTEP for Windows: an update. J. Appl. Crystallogr..

