# Supplementary material for: A diuranium carbide cluster stabilized inside a C80 fullerene cage
Source: Nat Commun. 2018 Jul 16;9:2753. doi: 10.1038/s41467-018-05210-8 (PMC6048043; doi:10.1038/s41467-018-05210-8)
Supplement: Supplementary file 1 — Supplementary Information [file 41467_2018_5210_MOESM1_ESM.pdf]

A Diuranium Carbide Cluster Stabilized Inside a C_{80} Fullerene Cage

Xingxing Zhang et al.

Supplementary Figures

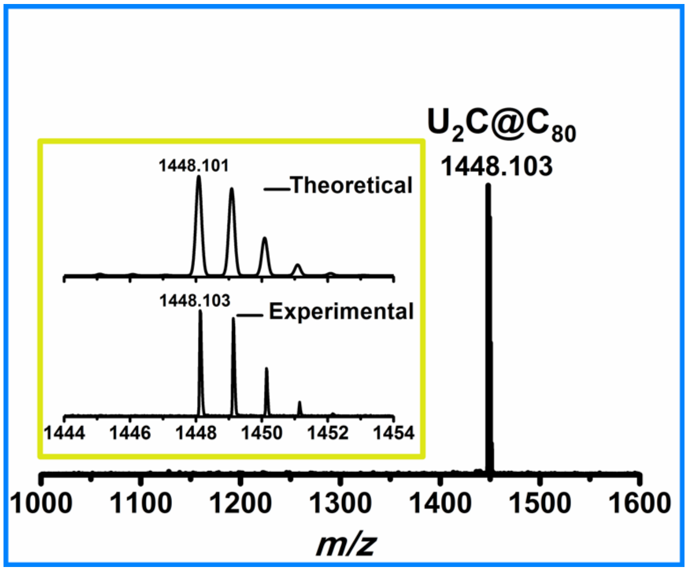

Supplementary Figure 1. Positive-ion mode MALDI-TOF mass spectrum of purified $U_2C@C_{80}$. Abscissa = Mass-number / Charge-number. Inset: experimental *vs.* theoretical isotopic distribution.

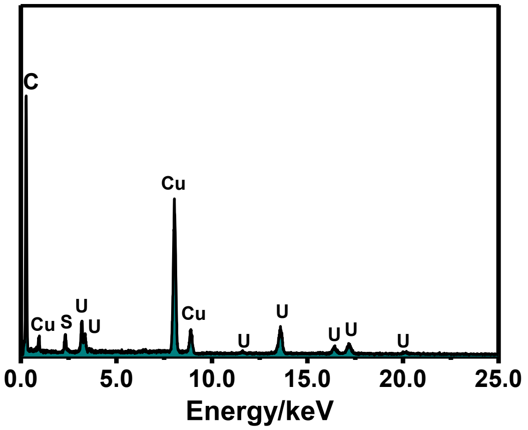

Supplementary Figure 2. EDS spectrum of the elemental composition of $U_2C@C_{80}$.

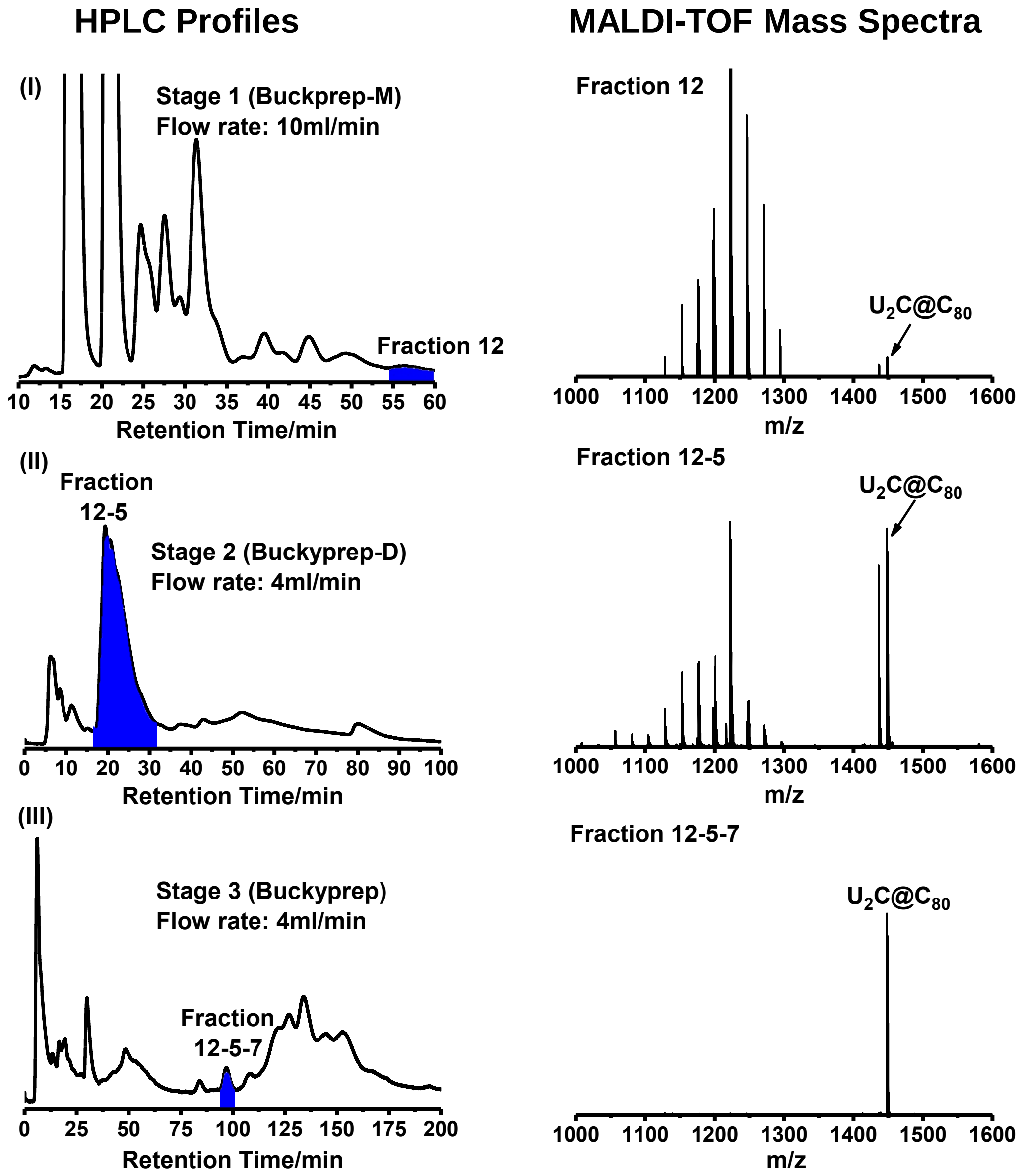

Supplementary Figure 3. Isolation scheme of $U_2C@C_{80}$. (I) *left.* The HPLC separation profile of the extracted mixture obtained by a modified arc-discharge synthesis (25×250 mm Buckyprep-M column; toluene as eluent; flow rate 10.0 mL/min; injection volume 9 mL). **(I)*right.*** The corresponding MALDI-TOF mass spectrum. **(II) *left.*** The second-step HPLC separation profile of fraction 12, collected from the first-step (10×250 mm Buckyprep-D column; toluene as eluent; flow rate 4.0 mL/min; injection volume 4 mL). **(II) *right.*** The corresponding MALDI-TOF mass spectrum. **(III) *left***. The third-step HPLC isolation profile of fraction 12-5, collected from the second-step (10×250 mm Buckyprep column; toluene as eluent; flow rate 4.0 mL/min; injection volume 4 mL). **(III) *right.*** The corresponding MALDI-TOF mass spectra. It shows that the purified $U_2C@I_h(7)$-C_{80} was obtained in fraction 12-5-7 (III *left*).

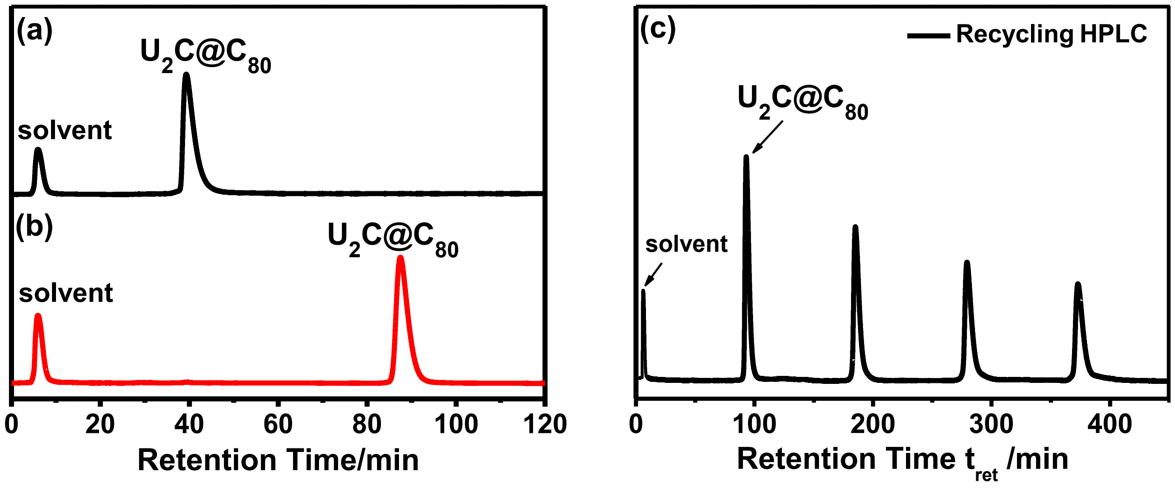

Supplementary Figure 4. HPLC chromatograms. **(a)** HPLC chromatograms of the purified $U_2C@C_{80}$ on a Buckyprep-M column with toluene as the eluent at the flow rate of 4.0 mL min^{-1} (black line). **(b)** HPLC chromatograms of the purified $U_2C@C_{80}$ on a Buckyprep column with toluene as the eluent at the flow rate of 4.0 mL min^{-1} (red line). **(c)** Recycling HPLC chromatogram of isolated $U_2C@C_{80}$ (10 × 250 mm Buckyprep column; flow rate 4.0 mL/min; injection volume 4 mL; toluene as eluent).

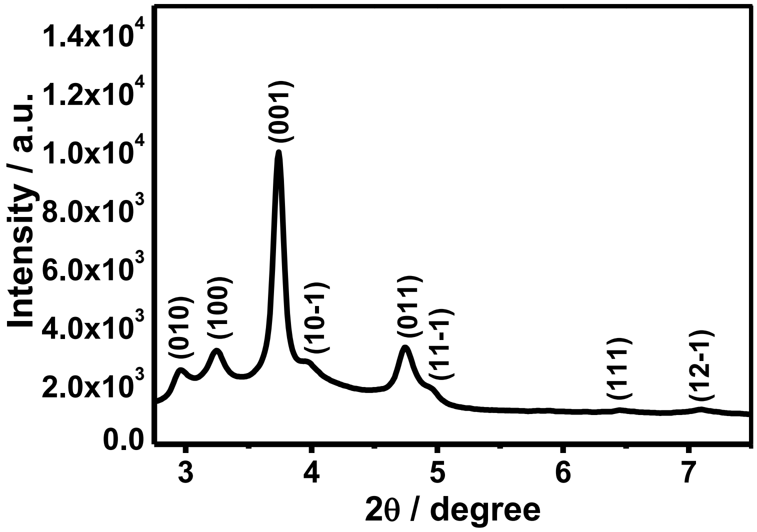

Supplementary Figure 5. Powder X-ray diffraction pattern of UCU@ I_h(7)-C_{80}. Peaks at 2θ = 2.93°, 3.21°, 3.70°, 3.92°, 4.70°, 4.88°, 6.39°, 7.03°, as observed at the Shanghai Synchrotron Radiation Facility (SSRF) at wavelength λ = 0.6199 Å, indexed as (010), (100), (001), (10-1), (011), (11-1), (111), (12-1), using the DIOPTAS[28] and DICVOL[29] programs for data integration and indexing, respectively. UCU@C_{80} shows a pure monoclinic phase with a = 10.3206 Å, b = 12.1510 Å, c = 11.8922 Å, β = 111.231° at ambient laboratory conditions.

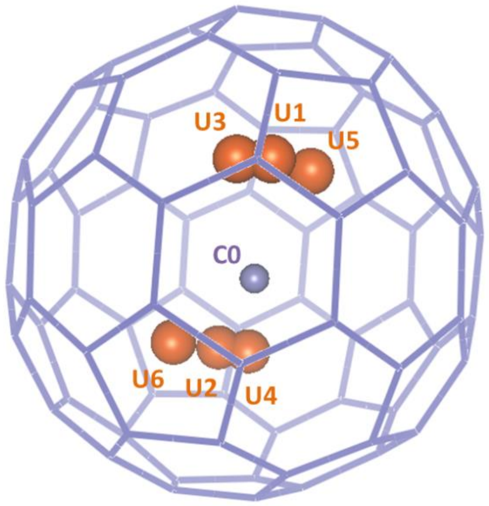

Supplementary Figure 6. View of X-ray structure of UCU@*I*$_h$(7)-C$_{80}$. It shows the U sites with occupancies, U1 and U2: 0.853(3), U3 and U4: 0.092(2), U5 and U6: 0.055(1). The sum of all U occupancies is 2.0, corresponding to U_2, while the I_h-C_{80} cage and the endohedral C atom (i.e., C0) have a common occupancy of 1.0, corresponding to C0@C_{80}.

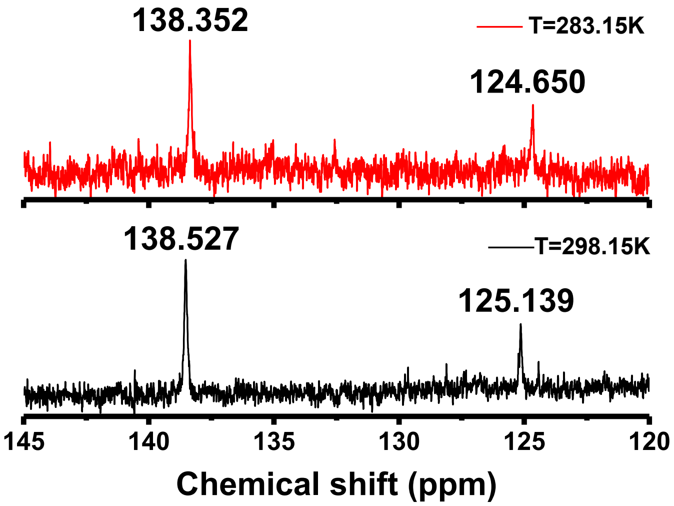

Supplementary Figure 7. **^{13}C nuclear magnetic resonance spectra of UCU@I_h(7)-C_{80}.**

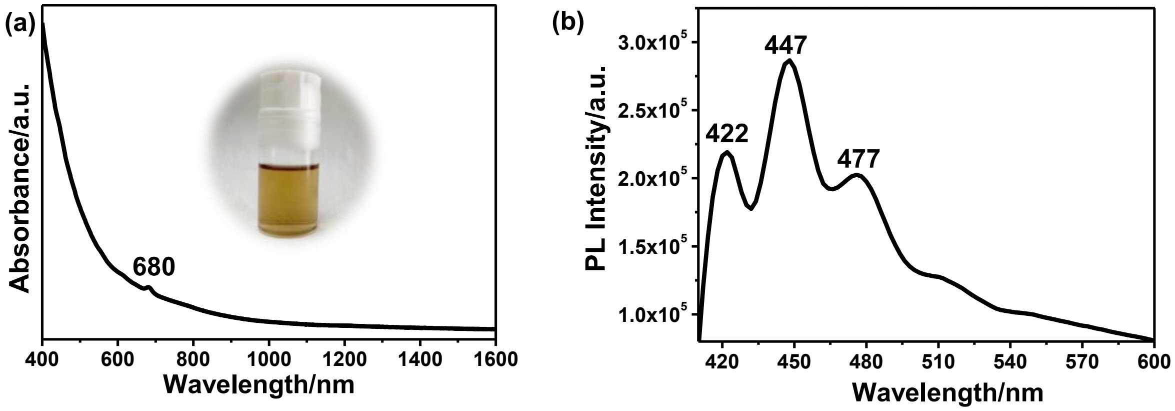

Supplementary Figure 8. UV-Vis-NIR and photo-luminescence spectra. **(a)** UV-Vis-NIR absorption spectrum of UCU@I_h(7)-C_{80} in CS_2. **(b)** Photo-luminescence spectrum of UCU@I_h(7)-C_{80} in CS_2 solution, upon excitation at 406 nm. Both spectra were recorded at room temperature.

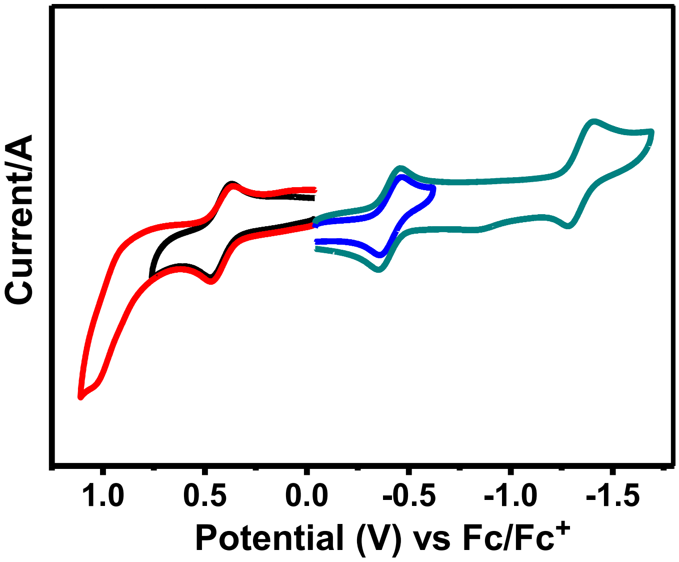

Supplementary Figure 9. Cyclic voltammogram of UCU@$\boldsymbol{I_h}$(7)-C_{80}. Solvent: *o*-dichlorobenzene (with 0.05 M (*n*-Bu)$_4$NPF$_6$). Scan rate 100 mV/s.

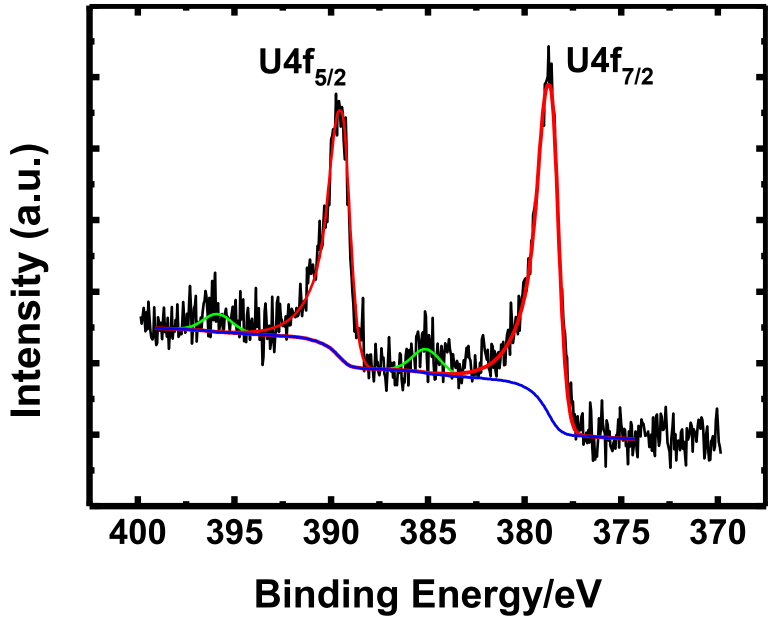

Supplementary Figure 10. XPS high-resolution spectrum of UCU@*I*h(7)-C80, in the U-4f region. The main U-4f doublet is shown in red whereas shake-up satellite peaks are shown in green. The Shirley background is indicated by a blue line.

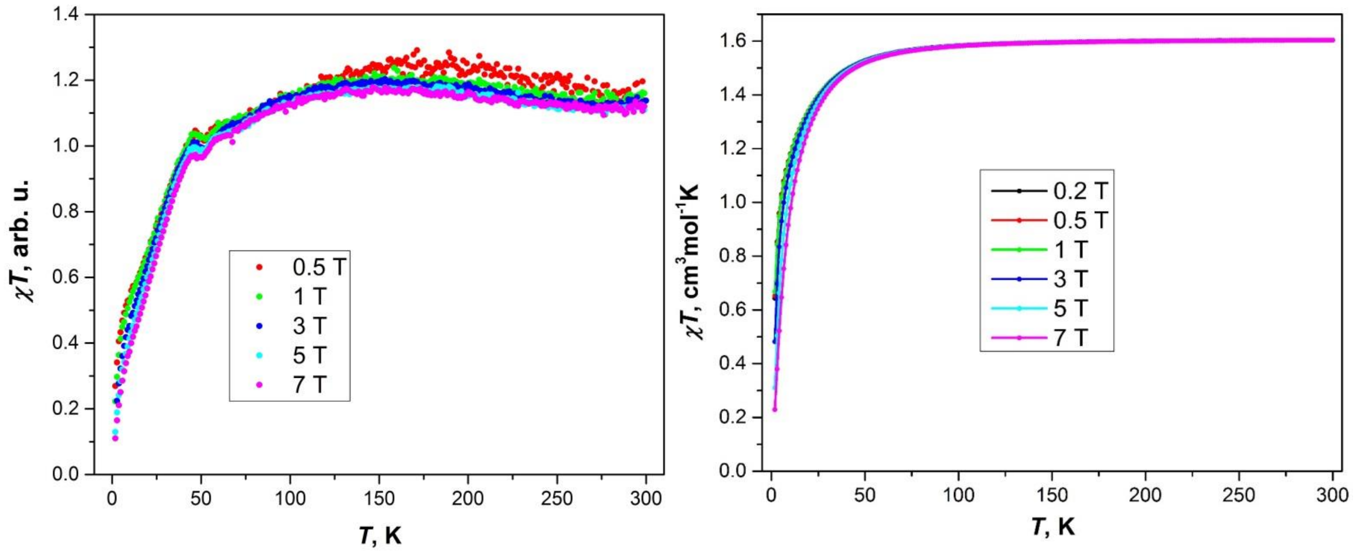

Supplementary Figure 11. Magnetism $T{\cdot}\chi$ of UCU@C_{80} *vs.* T (in K) in various magnetic fields. For temperatures from 1.8 to 300 K, and for magnetic flux densities up to 7 T.

Left: Measured. From the observed magnetization, an assumed temperature independent susceptibility background was subtracted, aimed at isolating the low temperature dependent paramagnetic contribution. By subtracting the feature just below 50 K (probably a contamination by solid O_2), smooth curves could be obtained. Due to the hypothetical background-correction and the small amount of substance, the absolute values for χT have large uncertainties, therefore the $cm^3mole^{-1}K$ units are named 'arbitrary units' as a precaution.

Right: Simulated. One of our many magnetic example simulations is shown, using a single-ion crystal (each U(f^1) with an m_J=5/2 ground state), the crystal field modelled by a single B_{20} term of 4 cm^{-1} (inducing easy-axis anisotropy) and an anti-ferromagnetic exchange coupling constant between the two U spins of −0.5 cm^{-1}. The experimental curves appear consistent with weak anisotropic anti-ferromagnetic coupling of two spins per molecule, somewhat coupled to orbital angular momenta. The experimentally deduced $T{\cdot}\chi$ (given in arbitrary units) appears to correspond to an effective molecular moment of the UCU@C_{80} molecules of a fraction of a μ_{Bohr} at the lowest temperatures, increasing at higher temperatures up to not more than 2 μ_{Bohr}.

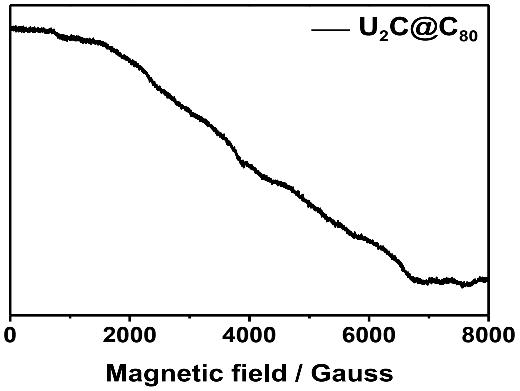

Supplementary Figure 12. X-Band EPR spectrum of UCU@I_h(7)-C_{80}. It is recorded in a toluene glass tube at 5 K (ν=9.368049 GHz, P = 9.464 mW, modulation = 2.0G at 100 kHz).

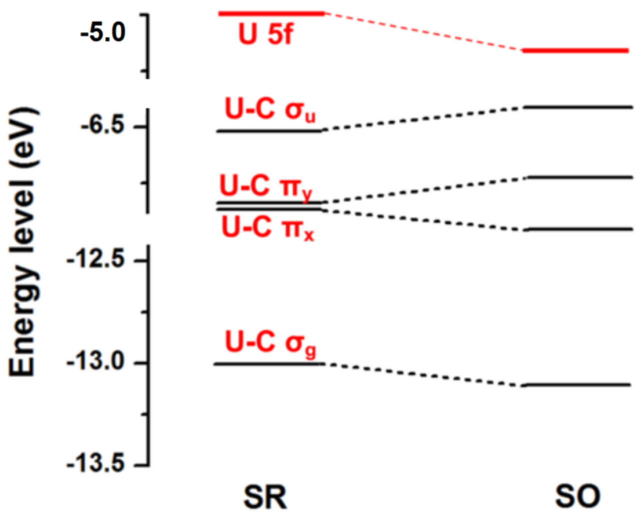

Supplementary Figure 13. Orbital energy levels (in eV) of the valence orbitals on the UCU unit of C_{2v}-UCU@$(I_3)_2$. The orbital is each occupied by 2 electrons, and calculated at the spin-averaged scalar-relativistic (SR) and spin-orbit (SO) coupled levels, from the PBE-ZORA relativistic density functional approximation.

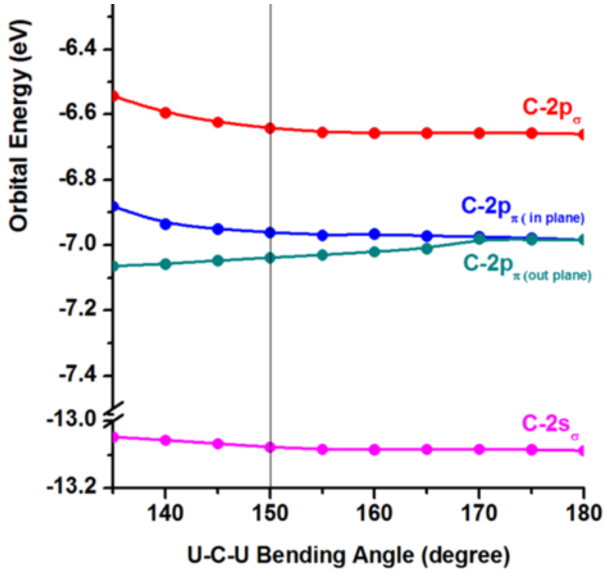

Supplementary Figure 14. Energies of the four UCU-bonding CMOs of $U_2C@(I_3)_2$ *vs.* bending angle of the UCU unit. Calculated from the PBE-ZORA relativistic density functional approximation. From 180º to 150º, the two UCU σ-binding MOs with ca. 70% C-2sp_z and 30% U_2-5f6d admixture increase very little, and the two UCU π-binding MOs with ca. 60% C-2$sp_{x,y}$ and 40% U-5f6d admixture decrease very little and split into an in-plane and a vertical component. The total molecular energy has a soft minimum near 150º bending. For stronger bending, the upper two valence CMOs start bending up, preventing smaller UCU angles.

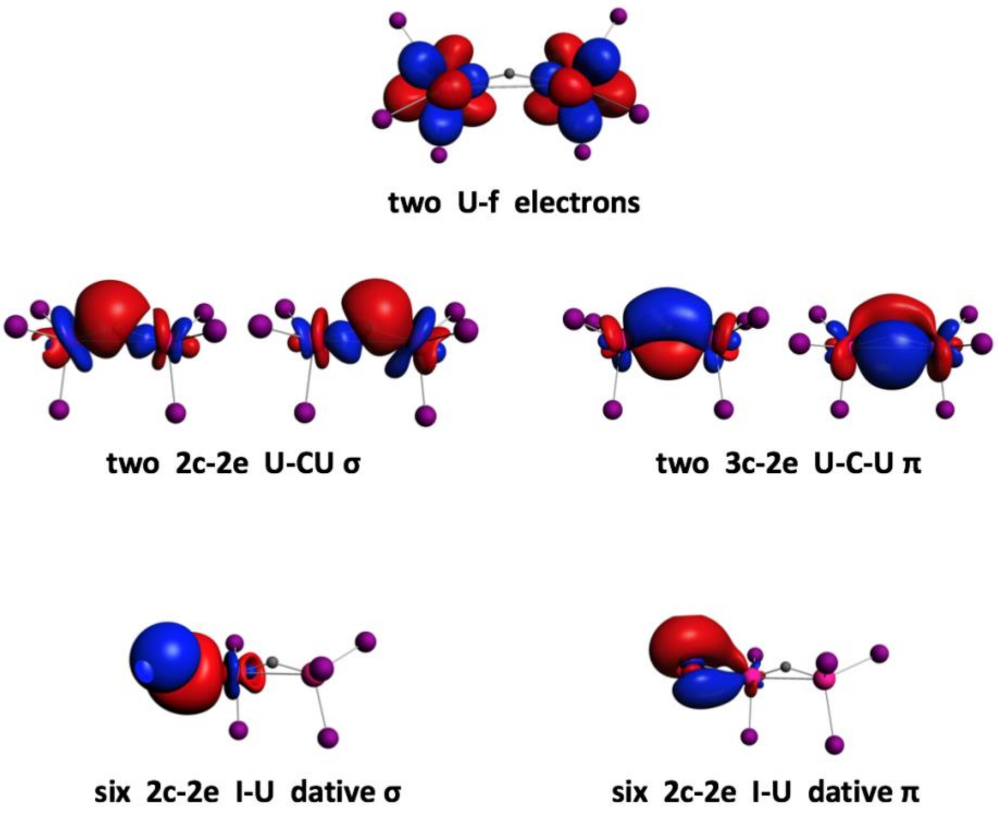

Supplementary Figure 15. Boys-localized valence MOs (LMOs) on the UCU of UCU@$(I_3)_2$. Calculated by the ZORA-PBE Kohn-Sham formalism. MO contour values are ± 0.03 (e/Å^3)^{½}. *Top:* Two U(5f)-type orbitals, each occupied by one electron.

Middle Left: Two U-C 2c-2e σ-pair LMOs of 69% C($2sp^1$), 24% U($5f6d^{0.7}$), 5% of the other U.

Middle Right: Two U-C 3c-2e π-pair LMOs of 57% C($2s^{0.3}p$) and 21% of each U($5f6d^{0.4}$).

Bottom: Dative 2c-2e σ-pair and π-pair LMOs of the I^{1-}-ligands into the U valence shells, with 14% σ-U($5f6d^{0.8}$) and 10% π-U($5f6d^1$), respectively.

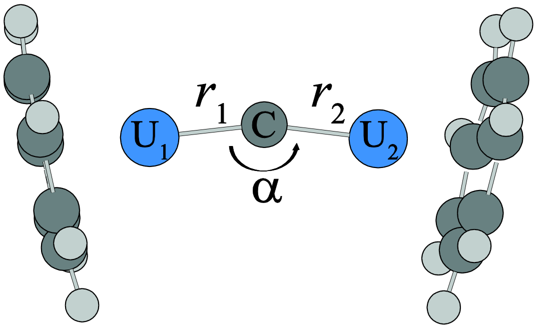

C_1 : r_1 = 2.08 Å, r_2 = 2.07 Å, α =166.1°

C_{2v}: r_1 = r_2 =2.08 Å, α =166.0°

Supplementary Figure 16: Sticks and Balls representation of (η^7-C_7H_7)UCU(η^7-C_7H_7).

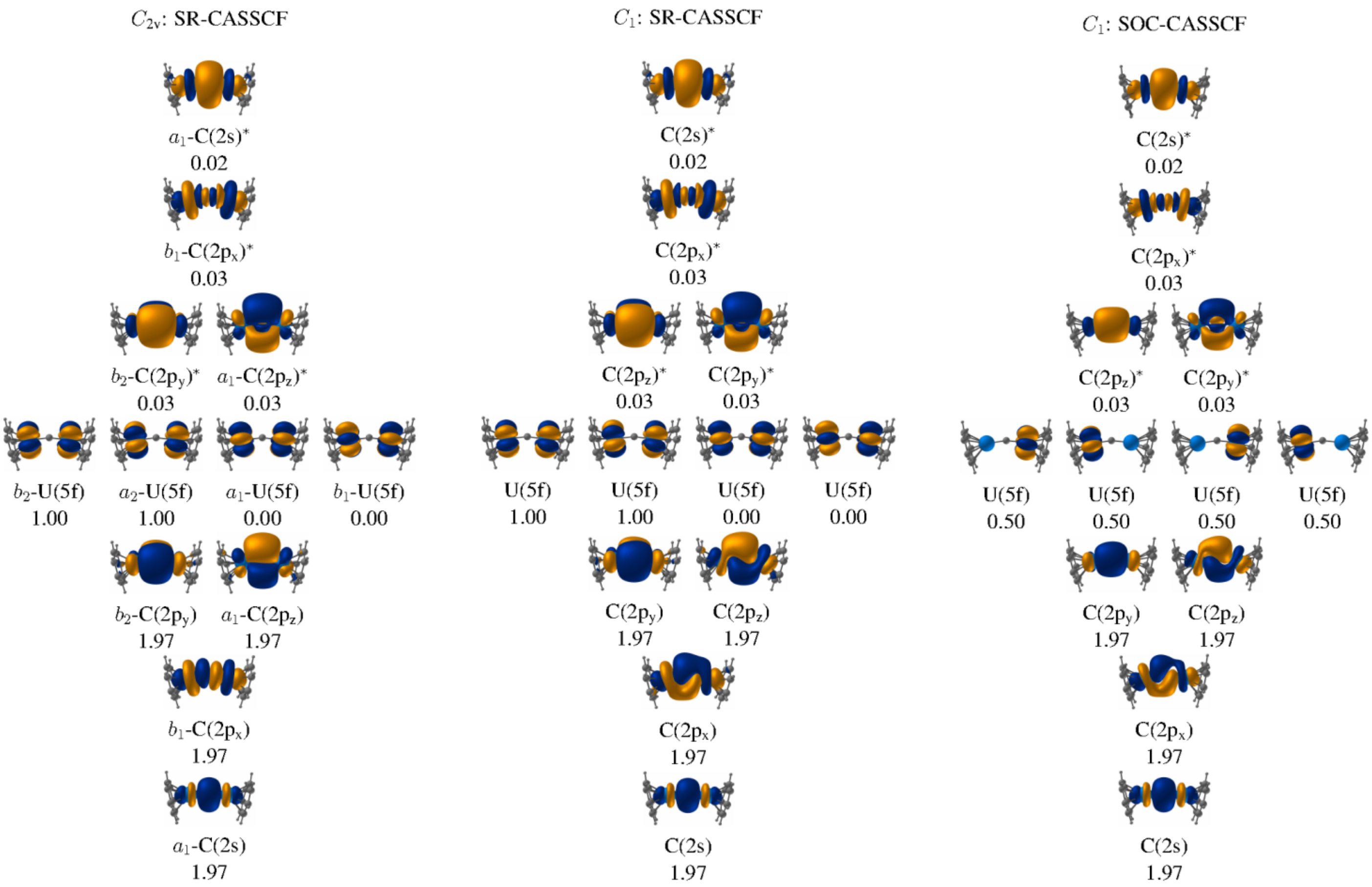

Supplementary Figure 17. Natural MOs of $(C_7H_7)UCU(C_7H_7)$. The isosurface values are $\pm0.042\sqrt{(e/Å^3)}$. Converged active space NOs with dominant AO contribution and occupation number for the lowest-lying states. *Left*: C_{2v} symmetry, scalar-relativistic, $^3B_1$. *Middle*: C_1 symmetry, scalar-relativistic, ^3A. *Right*: C_1 symmetry, with spin-orbit coupling, A.

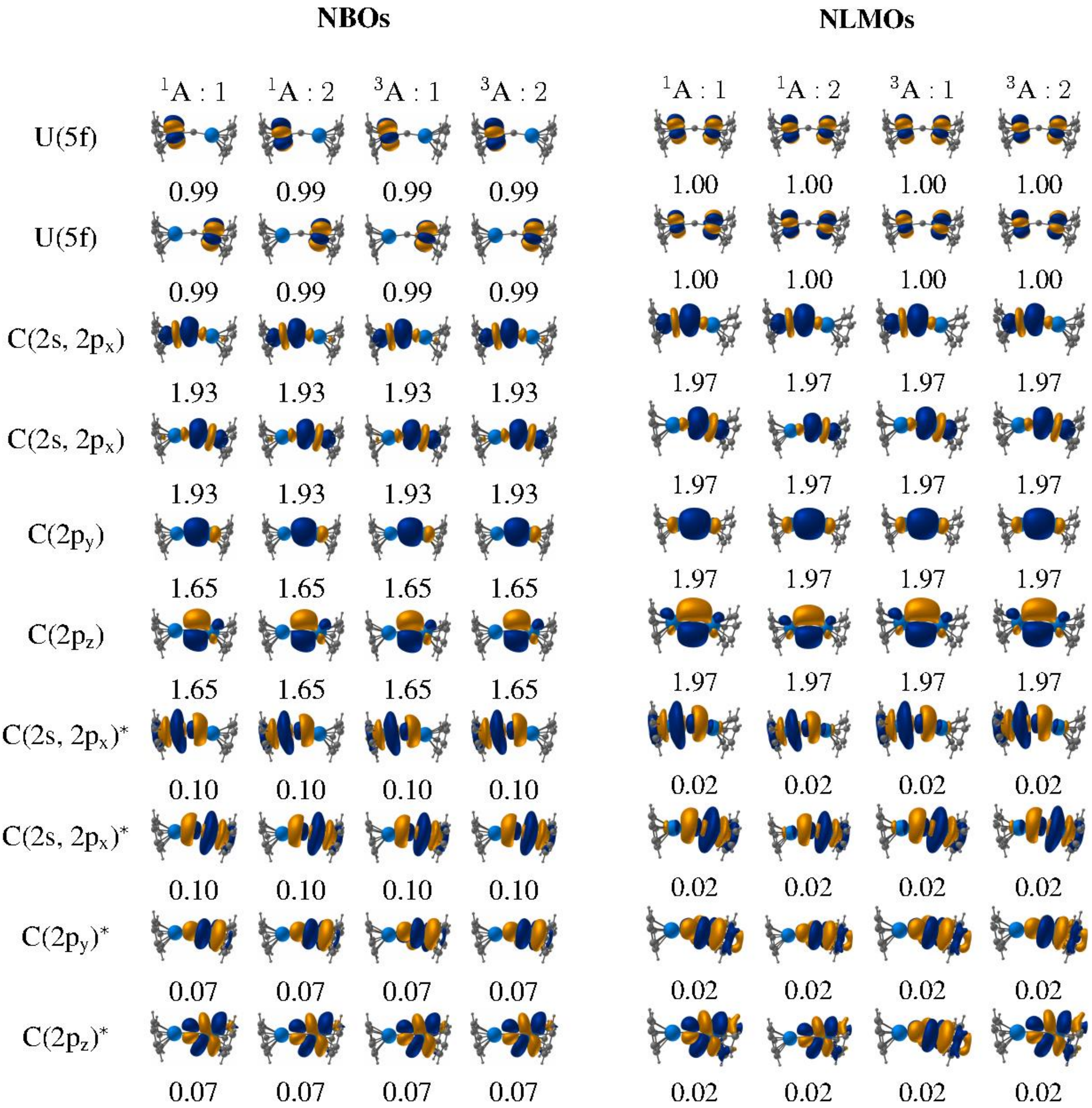

Supplementary Figure 18. Natural Localized Bond Orbital (NBO) and Natural Localized Molecular Orbital (NLMO) α,β spin-orbital-pair envelops of $(C_7H_7)UCU(C_7H_7)$, C_1 symmetry. Iso-surface values are ±0.042 $\sqrt{e/Å^3}$, with occupation numbers listed below, from SR-CASSCF calculations of the 2 lowest-lying ^1A and ^3A states (as listed in Supplementary Table 3).

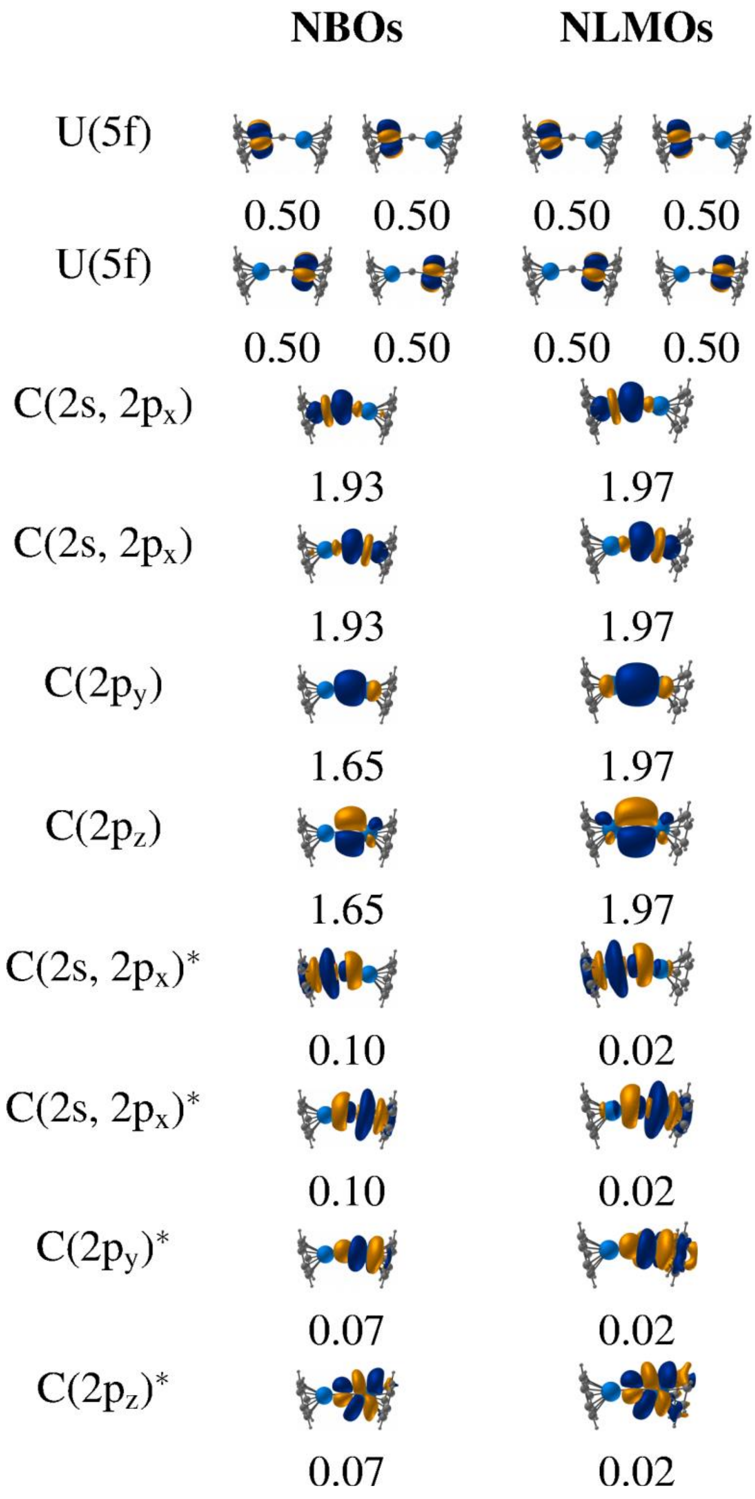

Supplementary Figure 19. Natural Localized Bond Orbital (NBO) and Natural Localized Molecular Orbital (NLMO) spinor-pair envelops of $(C_7H_7)UCU(C_7H_7)$ with C_1 symmetry. Iso-surface values are ±0.042 √e/Å^3, with occupation numbers listed below, from spin-orbit coupled calculations of the two lowest-lying states (as listed in Supplementary Table 4).

Supplementary Tables

Supplementary Table 1. Quantum-Chemical Effective Charges, Spins and Bond Orders.[a]

Atom	Property	Model a: $UCU@I_h(7)\text{-}C_{80}$	Model b: $UCU@(I_3)_2$	Model c: $UCU@(C_7H_7)_2$
**C_o**	Mulliken Charge	-1.0	-1.0	-1.0
	Hirshfeld Charge	-0.5	-0.5	-0.6
	Voronoi charge	-0.5	-0.4	-0.6
	Multipole charge	-0.9	-0.8	-1.8
	Bader Charge		-1.1	
	Mulliken Spin	-0.2		-0.15
	Mayer bonded valence	3.0		
**U**	Mulliken Charge	+0.3	+0.2	+1.0
	Hirshfeld Charge	+0.7	+0.5	+0.7
	Voronoi charge	+0.5	+0.5	+0.5
	Multipole charge	+1.6	+0.9	+1.9
	Bader Charge		+1.7	
	Mulliken Spin	+1.25		+1.2
	Mayer bonded valence	6.6		
	Mayer free valence	1.0		
**Outer Ligand**[b]	Mull. Charge	+0.35	+0.5	-1.0
	Mull.Ch. of each donating C	+0.2		+0.1
	Hirshfeld Charge of cage	-0.9	-0.5	
	Voronoi charge of cage	-0.5	-0.6	
	Mulliken Spin	-0.3		-0.3
	Mayer bonded valence	3.9 to 4.0		
	M.bond.val. of each donating C	ca. 3.6		
**MW Bond Orders** [c]				
	C=U	1.4	1.7	1.6
	U···U	-0.56	-0.66	-1.00
	Lig→U[d]	2*6*0.40	2*3*1.62	2*7*0.47
	Fullerene C-C	1.25±0.05		
	Donating C-C	1.06±0.02		

[a] From ZORA-PBE Kohn-Sham calculations. [b] Ligand = $(I_3)_2$ or C_{80} or $(C_7H_7)_2$. [c] MW-BO = Mayer-Wiberg bond order. [d] Donation from I or C atoms.

Supplementary Table 2. Typical interatomic distances [a] between U and main group elements C, N, O.

bonding	U–O	U–N	U–C
**single**	2.2 *(2.45)*	2.3 *(2.5)*	2.4 *(2.6)*
**double**	1.9	2.0 *(2.3)*	2.05 *(2.4)*
**triple**	1.8	1.85	1.9
**quadruple**			1.8

[a] Distances in Å, within ±5%. Axial ones *(equatorial ones in italics in parentheses)*. The interatomic distances of U significantly depend on the coordination number *CN* roughly as +0.075Å·*CN* (i.e. R. D. Shannon's ionic radii, *Acta Cryst. A* 1976, **32**, 751), on the formal charge *q* roughly as −0.1Å·*q*, and of course on crowding, on congestion and on strain of multidentate ligands. Also see the references in the main article.

Supplementary Table 3. 16 low-lying electronic states and relative energies of $(C_7H_7)UCU(C_7H_7)$.[a]

State (C_{2v})[b]	Conf.[c] ; weight (%)	E (eV)[d]	State (C_1)[e]	Conf. ; weight (%)	E (eV)
$^3B_1$	$(a_2)^1(b_2)^1$; 94	0.000 (0.132)	^3A	$(a)^1(a)^1$; 87	0.000
$^1A_1$	$(a_2)^2 + (b_2)^2$; 94	0.003 (0.124)	^1A	$(a)^1(a)^1$; 67	0.003
$^3A_2$	$(a_1)^1(a_2)^1 + (b_1)^1(b_2)^1$; 94	0.006 (0.019)	^3A	$(a)^1(a)^1$; 84	0.005
$^3B_2$	$(a_1)^1(b_2)^1 + (b_1)^1(a_2)^1$; 94	0.006 (0.019)	^3A	$(a)^1(a)^1$; 83	0.006
$^1A_2$	$(a_1)^1(a_2)^1 + (b_1)^1(b_2)^1$; 94	0.009 (0.001)	^1A	$(a)^1(a)^1$; 68	0.008
$^1B_2$	$(a_1)^1(b_2)^1 + (b_1)^1(a_2)^1$; 94	0.009 (0.000)	^1A	$(a)^1(a)^1$; 82	0.009
$^3B_1$	$(a_1)^1(b_1)^1$; 94	0.013 (0.147)	^3A	$(a)^1(a)^1$; 87	0.011
$^1A_1$	$(a_1)^2 + (b_1)^2$; 94	0.015 (0.139)	^1A	$(a)^1(a)^1$; 72	0.014

[a] Data from CASSCF calculations with scalar-relativistic approximation. [b] The C_{2v} geometry is used. [c] Given in terms of the nonbonding U(5f) MOs shown in Supplementary Figure 14. [d] CASPT2 values are given in parenthesis. [e] The C_1 geometry is used.

Supplementary Table 4. 8 Low-lying SOC electronic states and relative energies of $(C_7H_7)UCU(C_7H_7)$.[a]

CASSCF,[b] C_{2v}		CASPT2,[b] C_{2v}		CASSCF,[c] C_1	
SR composition	E (eV)	SR composition	E (eV)	SR composition[d]	E (eV)
50%$^3B_1$ + 49%$^3A_2$	0.000	53%$^1A_2$ + 46%$^3B_1$	0.000	98%^3A	0.000
50%$^3A_2$ + 49%$^3B_1$	0.000	54%$^3B_2$ + 45%$^1A_1$	0.006	98%^3A	0.000
50%$^3B_2$ + 48%$^1A_1$	0.001	53%$^3A_2$ + 46%$^3B_1$	0.009	40%^1A + 28%^3A	0.001
50%$^1A_1$ + 49%$^3B_2$	0.001	54%$^3A_2$ + 45%$^3B_1$	0.009	42%^3A + 28%^1A	0.001
100%$^3B_1$	0.754	97%$^1B_2$	0.687	100%^3A	0.754
100%$^3A_2$	0.754	97%$^3B_2$	0.704	100%^3A	0.754
97%$^1A_1$	0.757	100%$^3B_2$	0.705	97%^1A	0.757
100%$^1B_2$	0.757	100%$^3A_2$	0.705	97%^1A	0.757

[a] Data from SOC-CASSCF calculations. [b] The C_{2v} geometry is used. [c] The C_1 geometry is used. [d] Cumulated weights.

Supplementary Table 5. Cartesian coordinates of the model clusters.[a]

$(C_7H_7)UCU(C_7H_7)$

Cartesian Coordinates (in Å)

C_o	-0.0006	0.183061	0.308133
U1	2.058365	0.035836	0.099845
U2	-2.05935	0.079497	0.082359
C	-3.87738	-1.64194	0.445696
C	-4.19912	-0.552	1.310212
C	-4.32544	0.831307	0.986434
C	-4.16035	1.46889	-0.27947
C	-3.8291	0.881276	-1.53783
C	-3.58361	-0.4939	-1.83868
C	-3.60359	-1.61788	-0.95641
H	-3.7356	-2.60811	0.9364
H	-3.29094	-2.56901	-1.39441
H	-3.24985	-0.70135	-2.85849
H	-3.66636	1.582216	-2.36019
H	-4.20454	2.560938	-0.26708
H	-4.47899	1.501402	1.836098
H	-4.27445	-0.79727	2.372508
C	3.638295	-1.63154	-0.9383
C	3.588633	-0.51115	-1.82382
C	3.804055	0.869148	-1.52328
C	4.125275	1.469198	-0.26747
C	4.309072	0.835896	0.997506
C	4.21565	-0.55031	1.32257
C	3.916239	-1.64974	0.463087
H	3.338379	-2.5886	-1.37263
H	3.806311	-2.61927	0.955067
H	4.293976	-0.79182	2.385632
H	4.448439	1.51045	1.846154
H	4.151326	2.56172	-0.25785
H	3.613512	1.564676	-2.34446
H	3.265443	-0.72861	-2.8448

$(I_3)UCU(I_3)$

Cartesian Coordinates (in Å)

C_o	0	0	0.021054
U1	1.959089	0	-0.48771
U2	-1.95909	0	-0.48771
I	2.333339	0	-3.33849
I	-3.47197	-2.2966	0.386853
I	3.471969	-2.2966	0.386853
I	3.471969	2.2966	0.386853
I	-3.47197	2.2966	0.386853
I	-2.33334	0	-3.33849

UCU@I_h(7)-C_{80} Cartesian Coordinates (in Å)

C_o	-0.0005	-0.5334	0.006186
U1	1.957583	-0.02922	0.013719
U2	-1.95951	-0.03334	-0.00094
C	1.218465	0.745135	-3.84417
C	1.219003	-0.69194	-3.83948
C	-0.00301	1.482734	-3.80164
C	2.365131	1.184283	-3.11996
C	2.367492	-1.12888	-3.11608
C	0.001406	-1.43266	-3.79444
C	3.097511	0.026083	-2.64764
C	-1.22239	0.741431	-3.84374
C	-1.21851	-0.69552	-3.83885
C	-0.00494	2.71546	-3.09858
C	2.333262	2.370322	-2.3478
C	0.003136	-2.666	-3.09074
C	2.338216	-2.31594	-2.34146
C	1.156153	3.157941	-2.37702
C	1.164029	-3.10824	-2.37223
C	3.954615	0.022875	-1.49057
C	-2.3707	1.17714	-3.12011
C	-2.36591	-1.13594	-3.11595
C	3.119314	2.352075	-1.14575
C	-1.16762	3.154422	-2.3772
C	3.980585	1.257421	-0.72805
C	3.132931	-2.30553	-1.14589
C	-1.1567	-3.11192	-2.37247
C	-2.34244	2.363493	-2.34823
C	4.003169	-1.21433	-0.72938
C	-3.10039	0.016766	-2.64804
C	0.708699	3.874654	-1.2129
C	-2.33332	-2.32335	-2.34184
C	-0.72246	3.872485	-1.21301
C	0.72	-3.83385	-1.21285
C	2.64586	3.045068	0.006051
C	-0.71061	-3.83621	-1.21303
C	1.429194	3.805158	0.005898
C	2.655537	-2.99754	0.005406
C	3.982154	1.257548	0.74024
C	-3.12934	2.342954	-1.14666
C	4.005017	-1.21491	0.741016
C	-3.96018	0.01092	-1.49242
C	1.439583	-3.76259	0.005113

C	-3.12896	-2.31553	-1.14679
C	3.119791	2.352071	1.157746
C	-1.44309	3.800867	0.005526
C	3.958706	0.022488	1.503313
C	-3.98837	1.245796	-0.72942
C	3.133689	-2.30622	1.156921
C	-4.00295	-1.22664	-0.73069
C	-2.65743	3.037008	0.005234
C	-1.43069	-3.76706	0.004719
C	0.708371	3.874511	1.224468
C	-2.64896	-3.00573	0.004609
C	-0.7228	3.872333	1.22436
C	0.71974	-3.83433	1.222866
C	2.332928	2.370125	2.359292
C	-0.71086	-3.83641	1.222707
C	3.097977	0.025573	2.65843
C	-3.98724	1.245386	0.738938
C	1.155708	3.157477	2.38843
C	2.337985	-2.31679	2.351911
C	-3.12894	2.342424	1.15689
C	-4.00175	-1.22643	0.739619
C	-3.12835	-2.31524	1.156024
C	2.364803	1.183622	3.13085
C	1.163696	-3.10887	2.38237
C	2.366982	-1.1294	3.126203
C	-1.16812	3.153981	2.388272
C	-3.95787	0.010663	1.501472
C	-2.34289	2.362847	2.358911
C	-1.15709	-3.11228	2.382163
C	-0.00565	2.714851	3.109732
C	-2.33369	-2.32356	2.35157
C	0.002488	-2.66663	3.100744
C	1.21772	0.744315	3.854252
C	1.218172	-0.6926	3.849067
C	-3.10012	0.016195	2.658177
C	-0.00385	1.481946	3.812368
C	-2.37121	1.17651	3.130785
C	0.000515	-1.43342	3.804532
C	-2.36656	-1.13666	3.126354
C	-1.2231	0.740658	3.854668
C	-1.21928	-0.6964	3.849636

[a] Data from scalar-relativistic-ZORA PBE-Kohn-Sham calculations with valence-triple-zeta-polarized (TZP) STO type basis sets (ADF program suite).

Supplementary Methods

Mass, optical (UV-Vis-NIR, PL, Raman, FTIR) and NMR spectroscopies

A positive-ion mode matrix-assisted laser desorption/ionization time-of-flight facility (Bruker, Germany) was employed for the mass characterization. UV-Vis-NIR spectrum of purified UCU@I_h(7)-C_{80} was measured in CS_2 solution with a Cary 5000 spectrophotometer (Agilent, USA). The steady-state photoluminescence (PL) spectrum was recorded with an FLS980 spectrometer (Edinburgh Instrument, UK) by excitation at 406 nm at room temperature. The Raman spectrum was recorded on a Horiba Lab RAM HR Evolution Raman spectrometer using a laser at 633 nm. The Micro Fourier transform infrared spectrum was recorded at room temperature by a Vertex 70 spectrometer (Bruker, Germany) with a resolution of 4 cm^{-1}. For the IR and Raman measurements, the sample was drop-coated on aluminized paper and a quartz plate, respectively. The residual CS_2 was removed in a drying chamber in vacuum at 100 ℃. For the ^{13}C NMR spectroscopic measurements, the UCU@I_h(7)-C_{80} sample (ca.1.5 mg) was dissolved in CS_2 (0.8 mL) and placed into the NMR tube. A capillary containing acetone-D_6 was used as an internal lock. The ^{13}C NMR spectroscopic measurements were performed at 150 MHz (chemical shift measured in the range of -15 to 250 ppm) with an Agilent Direct-Drive II 600 MHz spectrometer (Agilent, USA) at 298 K.

Energy dispersive spectroscopy (EDS)

For the EDS analysis with a Transmission Electron Microscope (TEM), the purified sample was dispersed in an alcohol solution and then deposited on a TEM grid. The EDS spectrum was recorded on the TEM (FEI TECNAI G2 F20 200 kV) equipped with an EDS system (PV97-61700ME). The EDS spectrum shows characteristic peaks of uranium and carbon elements (Supplementary Figure 1a).

X-ray Photo-Electron Spectroscopy (XPS)

The XPS experiments were carried out in an ultra-high vacuum (UHV) chamber with a base pressure of $1 \cdot 10^{-10}$ mbar. The associated XPS set-up consists of a monochromatic X-ray source providing Al-Kα radiation ($\hbar\omega$ = 1486.6 eV) and a Phoibos 150 high resolution hemispherical electron analyzer (Specs, Germany) for photoelectron detection. UCU@I_h(7)-C_{80} droplets were deposited on Si supporting substrates. The angle between the substrate surface-normal and the detector was set to 35°. After subtraction of a Shirley background, the core levels of the main U-4f doublet were fitted with asymmetric approximated Voigt profiles. whereas the associated shake-up satellites were deconvoluted with symmetric approximated Voigt profiles. The spin-orbit splitting (U-4f 7/2 – 5/2) was set to 10.8 eV with an intensity ratio of 4:3 for both core levels and satellites.

Electrochemical Studies

Cyclic voltammetry (CV) and differential pulse voltammetry (DPV) were carried out in *o*-dichlorobenzene using a CHI-660E instrument. A conventional three-electrode cell consisting of a platinum counter electrode, a glassy carbon working electrode, and a silver reference electrode were used for both measurements. (n-Bu)$_4$NPF_6 (0.05 M) was used as supporting electrolyte. The CV and DPV curves were measured at scan rates of 100 and 20 mV/s, respectively.

Magnetometric Measurements

A sample of pristine solid UCU@I_h(7)-C_{80} (MW = 1448.8g) was prepared by drop-casting a carbon disulfide solution of ca. 0.1 mg, i.e. ca. 10^{-7} mol of UCU@C_{80}, mixed with polystyrene. Orientational motion of microcrystalline fullerene particles in strong magnetic fields may result in artifacts of the magnetic response, and encapsulating of the fullerene in the polymer matrix ensures that this is not happening. The magnetization was determined in magnetic fields of flux densities from 0.5 up to 7 Tesla, at temperatures T from 1.8 to 300 Kelvin, using an MPMS3 Vibrating Sample Magnetometer (VSM) with a sweeping rate of 2 K/min. We corrected for the expected diamagnetism of the fullerene and encapsulation-polymer. In addition, the significant temperature-independent background magnetism was tentatively subtracted, so that the low-temperature Curie-Weiss type temperature-dependent contribution to susceptibility, χ_{CW}, was obtained. The resulting product $T \cdot \chi_{CW}$ *vs.* T curves are plotted in Supplementary Figure 9, together with theoretical simulations.

EPR Measurements

Attempts to further resolve the electronic structure of UCU@I_h(7)-C_{80} using EPR spectroscopy. Samples was dissolved in a toluene solution with about 0.5 mg/ml and then added into a quartz tube for EPR experiments at 5 K. The EPR spectrum was measured on a Bruker Elexsys E580 spectrometer. No clearly defined signal was observed, see Supplementary Figure 8. Factors such as nuclear quadrupole couplings, near degenerate electronic states and cage shielding effects complicate the analysis. Thus, additional studies of the electronic properties of this unique and unprecedented system is warranted and will be communicated in due time.

Quantum Computational Methods

Quantum chemical calculations were performed with theories at different levels of sophistication.

(a) Quasi-relativistic density-functional approaches

The program codes ADF2016.101, Gaussian09, and ORCA4.0 were applied for the electronic SCF and nuclear geometry optimizations. Bonding analyses were then performed using the NBO5.0 and Multiwfn codes[1-6]. In order to improve the convergence of the two-open-shells singlet state, the two-orbital-mixing approach was applied at the spin-averaged spin-unrestricted Kohn-Sham level of approximation. Relativity in the U atoms was accounted for either by scalar-relativistic or by spin-orbit coupled effective small-core pseudopotentials or by the correspondingly frozen atomic-core zero-order regular approach[7,8]. Perdew-Burke-Ernzerhoff's density functional was applied, either the simple gradient correct one (PBE) or the exchange-hybrid one (PBE0) without or with the dispersion-correction by the Becke-Johnson damping scheme (D3BJ)[9-12]. Basis sets of Gaussian cc-pVTZ or of Slater triple-valence-zeta polarized type were used as supplied in the codes. The geometry optimizations and frequency calculations conducted with ORCA were performed with the ZORA-def2-SVP for the carbon atoms (exponents from the def2-SVP basis set recontracted for ZORA and the SARC-ZORA-TZVP basis for the uranium atoms[13-15]. The single point energy calculations conducted with ORCA were performed with either the ZORA-SV(P) (for C) and SARC-ZORA-TZVP (for U) basis sets (bond order analyses and generation of the wfn file) or ZORA-def2-TZVP (for C) and SARC-ZORA-TZVPP (for U) basis sets (orbital energies and gaps).

(b) Quasi-relativistic correlated *ab initio* approaches

Ab initio wave-function-based calculations were performed, using a developer's version of the Molcas package (version 8.1)[16], to get insights on the electronic structure and bonding in the (η^7-C_7H_7)UCU(η^7-C_7H_7) system. Scalar relativistic (SR) wave functions for the lowest-lying spin-singlet and spin-triplet electronic states were obtained through state specific (SS) and/or state averaged (SA) complete active space self-consistent field (CASSCF)[17] calculations. The C_1 spin-triplet ground-state (GS) equilibrium structure, optimized using DFT/PBE, was considered as well as a C_{2v} symmetrized version of it (Supplementary Figure 13). The Douglas-Kroll-Hess Hamiltonian[19–21] was used alongside ANO-RCC basis sets of valence triple-ζ quality[22–25]. The active space correlated 10 electrons in 12 molecular orbitals (MOs), i.e. CASSCF(10, 12). In C_{2v} symmetry, five active MOs span the a_1 irreducible representation (irrep): two bonding and their antibonding pairs with C(2s) and C($2p_z$) characters plus a nonbonding U(5f) orbital; three active MOs span the b_1 irrep: the bonding and antibonding pair with C($2p_x$) character plus a nonbonding U(5f) orbital; one active MO spans the a_2 irrep: a nonbonding U(5f) orbital; three active MOs span the b_2 irrep: the bonding and antibonding MOs with C($2p_y$) character plus a nonbonding U(5f) orbital. The four nonbonding 5f orbitals are formally degenerate pairs, U_1-and U_2-based, with isosurface contours similar to those of 5f orbitals in linear symmetry. 2 electrons in 4 orbitals give $8\times7/1\times2 = 28$ states, at the scalar level 10 singlets and 6 triplets.

16 states of 8 electronic scalar (SR) levels were calculated, 4 formally degenerate spin-triplet ones: $2\times{}^3B_1$, $1\times{}^3A_2$, $1\times{}^3B_2$, and 4 formally degenerate spin-singlet ones: $2\times{}^1A_1$, $1\times{}^1A_2$, $1\times{}^1B_2$. The same active space was used, and the same electronic states were calculated, in CASSCF calculations with C_{2v} and C_1 symmetry. Additional dynamic correlation was introduced with the complete active space perturbation theory at second order (CASPT2) method[18]. In CASPT2 calculations, the default imaginary shift (0.20) and ionization potential electron affinity shift (0.25) were used. The SR wave functions were then used to build a state-interaction (SI) matrix for the spin orbit coupling (SOC) operator, whose diagonal was dressed with either CASSCF or CASPT2 correlated energies. The diagonallization of this matrix yielded SOC corrected electronic states and energies. The electronic structure and bonding in the (η^7-C_7H_7)UCU(η^7-C_7H_7) complex was further scrutinized from the view point of localized orbitals. Natural bond orbital (NBO) calculations were performed, using the CASSCF and SO-CASSCF densities, with a locally developed program[26] interfacing Molcas with NBO6.[27] Plots of NBOs and natural localized molecular orbitals (NLMOs) were also provided.

Discussion: Calculated low-lying electronic states for the (η^7-C_7H_7)UCU(η^7-C_7H_7) complex are listed in Supplementary Tables 3 and 4 while converged sets of active space NOs, and their occupation numbers before and after the treatment of SOC, are shown in Supplementary Figure 14. The bonding (and antibonding) NOs involve the 2s and 2p valence shells of the C atom and valence U 5f and 6d shells. The two unpaired electrons are distributed among the four, U_1 and U_2 5f-based, nearly degenerate nonbonding NOs giving rise to a pool of low-lying and closely spaced electronic states. The SR-CASSCF wave-function compositions and relative energies listed in Supplementary Table 3 stand for a nearly eightfold degenerate electronic ground state. In both C_{2v} and C_1 symmetry groups, the SR ground state is spin-triplet (${}^3B_1$ and ${}^3A_1$ in C_{2v}), separated by only a few meV from the remaining computed states. As imposed by the point group, the most noticeable energy gaps in C_{2v} occur between the two SA states in ${}^3B_1$ symmetry and between the lowest ${}^3B_1$ state and the two SA states in ${}^1A_1$ symmetry. A similar qualitative and quantitative trend is present for the corresponding states in the C_1 point group (Supplementary Table 3). The additional dynamic correlation, introduced through CASPT2 calculations with C_{2v} symmetry constraints, favors a nearly degenerate spin-singlet ground state, ${}^1B_2$ and ${}^1A_2$, separated to a slightly larger extent from the remaining computed states, by 0.02 eV from the ${}^3A_2$ and ${}^3B_2$ states, and by 0.13 eV from the sets of ${}^3B_1$ and ${}^1A_2$ states. These energy

gaps may however be slightly overestimated due to slight artificial symmetry breaking induced by CASPT2. Note that in an ideal linear symmetry, the calculated eight electronic states should be exactly degenerate.

When SOC is accounted for (Supplementary Table 4), CASSCF predicts an essentially spin-triplet SOC electronic ground state, parented in the spin components of the $^3B_1$ and $^3A_2$ SR states when C_{2v} symmetry constraints are used. Relaxing these constraints, a similar SOC ground state is obtained although the wave function has admixtures from spin components of all the coupled SR spin-triplet states. The additional dynamic correlation, projected on the diagonal of the SOC matrix in C_{2v} calculations (see CASPT2 values in Supplementary Table 4), favors a large admixture (53%) of the $^1A_2$ spin component in the SOC ground state. That is, the ground state in SO-CASPT2 calculations is predicted to be a nearly equal spin-singlet – spin-triplet mixture.

Irrespective of the nature of the electronic ground state, point group symmetry or formalism (SR or SOC), the bonding picture in (η^7-C_7H_7)UCU(η^7-C_7H_7) is unchanged. That is, since all the Slater determinants that build up the eight nearly-degenerate states only differ in the occupations of the nonbonding U(5f) NOs (i.e. the occupations of the remaining NOs are nearly identical for all calculated electronic states). For the lowest-lying SR state, active space NOs and natural occupation numbers obtained in SR-CASSCF calculations with C_{2v} and C_1 symmetry constraints, respectively, are shown in Supplementary Figure 14. Notably, the NOs show that the U–C–U backbone is maintained by two three-center bonds, each of them bearing a formal bond order of two, considering a single determinant formalism (Hartree-Fock or density DFT). In a multi-determinant CASSCF one, from the occupations of the bonding and nonbonding NOs (Supplementary Figure 14) an effective bond order (EBO) of 1.94 is obtained. Obviously, since SOC leads to admixtures of spin-components of SR states that exhibit similar EBOs, the SOC-EBO of any calculated SOC state is 1.94. The example of the ground SOC state (obtained through SOC-CASSCF) is shown in Supplementary Figure 14.

In terms of localized orbitals, the bonding picture in the (η^7-C_7H_7)UCU(η^7-C_7H_7) complex predicted by NBO calculations is in full agreement with the one described above (in terms of (SOC-)CASSCF NOs). Plots of NBOs and NLMOs corresponding to the active-space NOs (reported in Supplementary Figure 14) are displayed in Supplementary Figure 15, for the two lowest-lying spin-singlet and spin-triplet states obtained in CASSCF calculations (C_1 symmetry), and in Supplementary Figure 16, for the lowest-lying SOC state displayed in Supplementary Table 4 (C_1 symmetry). The results strengthen the fact that the U–C–U back-bone is maintained by two three-center bonds. The calculated (SOC-)EBO, for each bond, using the natural occupations of the NLMOs, is 1.95, in full agreement with the EBO predicted by (SOC-CASSCF), 1.94.

Supplementary References

1. Baerends, E. J. et al. ADF, 2016.101 (SCM, Theoretical Chemistry, Vrijie Universiteit, Amsterdam 2016).
2. Frisch, M. J. et al. *Gaussian 03, revision D.02* (Gaussian, Inc., Wallingford, CT, 2004).
3. Neese, F. The ORCA program system. *WIRES. Comput. Mol. Sci.* **2,** 73-78 (2012).
4. Neese, F. et al. *ORCA – an ab initio, density functional and semiempirical program package, V.4.0.0* (MPI für Chemische Energiekonversion, Mülheim a. d. Ruhr, 2017).
5. Weinhold, F. & Landis, C. R. *Valency and Bonding. A Natural Bond Orbital Donor-Acceptor Perspective* (Cambridge University Press, Cambridge, UK, 2005).
6. Lu, T. & Chen, F. Multiwfn: A multifunctional wavefunction analyzer. *J. Comput. Chem.* **33,** 580-592 (2012).
7. Dolg, M. & Cao, X. Accurate relativistic small-core pseudopotentials for actinides. Energy adjustment for uranium and first applications to uranium hydride. *J. Phys. Chem. A* **113,** 12573-12581 (2009).
8. Lenthe, E. V., Baer1ends, E. J. & Snijders, J. G. Relativistic regular two-component Hamiltonians. *J. Chem. Phys.* **99,** 4597-4610 (1993).
9. Perdew, J. P., Burke, K. & Ernzerhof, M. Generalized gradient approximation made simple. *Phys. Rev. Lett.* **77,** 3865-3868 (1996).
10. Adamo, C. Barone, V. Toward reliable density functional methods without adjustable parameters: The PBE0 model. *J. Chem. Phys.* **110,** 6158-6170 (1999).
11. Grimme, S., Ehrlich, S. & Goerigk, L. Effect of the damping function in dispersion corrected density functional theory. *J. Comput. Chem.* **32,** 1456-1465 (2011).
12. Becke, A. D. & Johnson, E. R. A density-functional model of the dispersion interaction. *J. Chem. Phys.* **123,** 154101 (2005).
13. Dunning, T. H. Gaussian basis sets for use in correlated molecular calculations. I. The atoms boron through neon and hydrogen. *J. Chem. Phys.* **90,** 1007-1023 (1989).
14. Weigend, F. & Ahlrichs, R. Balanced basis sets of split valence, triple zeta valence and quadruple zeta valence quality for H to Rn: Design and assessment of accuracy. *Phys. Chem. Chem. Phys.* **7,** 3297-3305 (2005).
15. Pantazis, D. & Neese, A. F. All-electron scalar relativistic basis sets for the actinides. *J. Chem. Theory Comput.* **7,** 677-684 (2011).
16. Aquilante, F. et al. Molcas 8: New capabilities for multiconfigurational quantum chemical calculations across the periodic table. *J. Comput. Chem.* **37,** 506-541 (2016).
17. Roos, B. O., Taylor, P. R. & Siegbahn, P. E. M. A complete active space SCF method (CASSCF) using a density matrix formulated super-CI approach. *Chem. Phys.* **48,** 157-173 (1980).
18. Andersson, K., Malmqvist, P. A., Roos, B. O., Sadlej, A. J. & Wolinski, K. Second-order perturbation theory with a CASSCF reference function. *J. Phys. Chem.* **94,** 5483-5488 (1990).
19. Douglas, M. & Kroll, N. M. Quantum electrodynamical corrections to the fine structure of helium. *Ann. Phys.* **82,** 89-155 (1974).
20. Hess, B. A. Applicability of the no-pair equation with free-particle projection operators to atomic and molecular structure calculations. *Phys. Rev. A* **32,** 756-763 (1985).
21. Hess, B. A. Relativistic electronic-structure calculations employing a two-component no-pair formalism with external-field projection operators. *Phys. Rev. A* **33,** 3742-3748 (1986). Wolf, A., Reiher, M. & Hess, B. A. *J. Chem. Phys.***117**, 9215-9226 (2002).

22. Widmark, P.-O., Malmqvist, P.-Å. & Roos, B. O. Density matrix averaged atomic natural orbital (ANO) basis sets for correlated molecular wave functions. *Theor. chim. Acta* **77,** 291-306 (1990).
23. Roos, B. O., Lindh, R., Malmqvist, P.-Å., Veryazov, V. & Widmark, P.-O. Main group atoms and dimers studied with a new relativistic ANO basis set. *J. Phys. Chem. A* **108,** 2851-2858 (2004).
24. Roos, B. O., Lindh, R., Malmqvist, P.-Å., Veryazov, V. & Widmark, P.-O. New relativistic ANO basis sets for actinide atoms. *Chem. Phys. Lett.* **409,** 295-299 (2005).
25. Malmqvist, P. Å., Roos, B. O. & Schimmelpfennig, B. The restricted active space (RAS) state interaction approach with spin–orbit coupling. *Chem. Phys. Lett.* **357,** 230-240 (2002).
26. Duignan, T. J. & Marchenko, A. Exatomic: A unified platform for computational chemists. https://github.com/exa-analytics/exatomic, doi:10.5281/zenodo.60053.
27. Glendening, E. D., Landis, C. R. & Weinhold, F. NBO 6.0: Natural bond orbital analysis program. *J. Comput. Chem.* **34,** 1429-1437 (2013).
28. Prescher, C. & Prakapenka, V. B. DIOPTAS: a program for reduction of two-dimensional X-ray diffraction data and data exploration. *High Pressure Res.* **35**, 223-230 (2015).
29. Louër, D. & Boultif, A. Indexing with the successive dichotomy method, DICVOL04. *Z. Kristallogr. Suppl.* **23**, 225-230 (2006).
