# Supplementary material for: A diuranium carbide cluster stabilized inside a C80 fullerene cage
Source: Nat Commun. 2018 Jul 16;9:2753. doi: 10.1038/s41467-018-05210-8 (PMC6048043; doi:10.1038/s41467-018-05210-8)
Supplement: Supplementary file 3 — Description of Additional Supplementary Files [file 41467_2018_5210_MOESM3_ESM.pdf]

Description of Additional Supplementary Files

File Name: Supplementary Data 1
Description: CIF file for the single-crystal X-ray diffraction data of UCU@Ih-C80·[NiII -OEP](1).

File Name: Supplementary Data 2
Description: Checkcif file for single-crystal X-ray diffraction data of the UCU@Ih-C80·[NiII -OEP](1).
