# Supplementary material for: A diuranium carbide cluster stabilized inside a C80 fullerene cage
Source: Nat Commun. 2018 Jul 16;9:2753. doi: 10.1038/s41467-018-05210-8 (PMC6048043; doi:10.1038/s41467-018-05210-8)

checkCIF/PLATON report

Structure factors have been supplied for datablock(s) shelx

THIS REPORT IS FOR GUIDANCE ONLY. IF USED AS PART OF A REVIEW PROCEDURE FOR PUBLICATION, IT SHOULD NOT REPLACE THE EXPERTISE OF AN EXPERIENCED CRYSTALLOGRAPHIC REFEREE.

No syntax errors found. CIF dictionary Interpreting this report

Datablock: shelx

Bond precision:	C-C = 0.0076 A		Wavelength=0.71073
Cell:	a=17.678(4)	b=16.970(3)	c=26.695(5)
	alpha=90	beta=106.65(3)	gamma=90
Temperature:	120 K		

	Calculated	Reported
Volume	7673(3)	7673(3)
Space group	P 21/c	P 21/c
Hall group	-P 2ybc	-P 2ybc
Moiety formula	C81 U2, C36 H44 N4 Ni, 1.5(C6 H6), C S2	U2C C80, Ni N4 C36 H44, C9 H9, C S2
Sum formula	C127 H53 N4 Ni S2 U2	C127 H53 N4 Ni S2 U2
Mr	2233.70	2233.62
Dx,g cm-3	1.934	1.934
Z	4	4
Mu (mm-1)	4.578	4.578
F000	4348.1	4348.0
F000’	4274.86	
h,k,lmax	22,21,34	22,21,34
Nref	16914	16771
Tmin,Tmax	0.795,0.913	0.558,0.746
Tmin’	0.795	

Correction method= # Reported T Limits: Tmin=0.558 Tmax=0.746
AbsCorr = MULTI-SCAN

Data completeness= 0.992 Theta(max)= 27.092

R(reflections)= 0.0388(13046) wR2(reflections)= 0.1045(16771)

S = 1.007 Npar= 1278

The following ALERTS were generated. Each ALERT has the format
test-name_ALERT_alert-type_alert-level.
Click on the hyperlinks for more details of the test.

Alert level A

PLAT307_ALERT_2_A Isolated Metal Atom found in Structure (Unusual) U01 Check

Author Response: This is an endohedral metal atom.

PLAT307_ALERT_2_A Isolated Metal Atom found in Structure (Unusual) U02 Check

Author Response: This is an endohedral metal atom.

PLAT307_ALERT_2_A Isolated Metal Atom found in Structure (Unusual) U03 Check

Author Response: This is an endohedral metal atom.

PLAT307_ALERT_2_A Isolated Metal Atom found in Structure (Unusual) U04 Check

Author Response: This is an endohedral metal atom.

PLAT307_ALERT_2_A Isolated Metal Atom found in Structure (Unusual) U05 Check

Author Response: This is an endohedral metal atom.

PLAT307_ALERT_2_A Isolated Metal Atom found in Structure (Unusual) U06 Check

Author Response: This is an endohedral metal atom.

Alert level B

PLAT910_ALERT_3_B Missing # of FCF Reflection(s) Below Theta(Min). 19 Note

Author Response: Some reflections below theta(min) are too strong, and therefore these reflections were excluded.

Alert level C

```
PLAT147_ALERT_1_C s.u. on Symmetry Constrained Cell Angle(s) .....     Please Check
PLAT213_ALERT_2_C Atom U04            has ADP max/min Ratio .....        3.1 prolat
PLAT213_ALERT_2_C Atom C26A           has ADP max/min Ratio .....        3.5 oblate
PLAT213_ALERT_2_C Atom C29A           has ADP max/min Ratio .....        3.3 oblate
PLAT213_ALERT_2_C Atom C35A           has ADP max/min Ratio .....        3.2 oblate
PLAT213_ALERT_2_C Atom C36A           has ADP max/min Ratio .....        3.7 oblate
PLAT213_ALERT_2_C Atom C46A           has ADP max/min Ratio .....        3.4 oblate
PLAT213_ALERT_2_C Atom C56A           has ADP max/min Ratio .....        3.3 oblate
PLAT213_ALERT_2_C Atom C59A           has ADP max/min Ratio .....        3.6 prolat
PLAT213_ALERT_2_C Atom C60A           has ADP max/min Ratio .....        3.6 prolat
```

```
PLAT213_ALERT_2_C Atom C61A           has ADP max/min Ratio .....        3.2 oblate
PLAT213_ALERT_2_C Atom C67A           has ADP max/min Ratio .....        3.3 oblate
PLAT213_ALERT_2_C Atom C75A           has ADP max/min Ratio .....        3.1 oblate
PLAT220_ALERT_2_C Non-Solvent Resd 1   U   Ueq(max)/Ueq(min) Range       4.4 Ratio
PLAT223_ALERT_4_C Solv./Anion Resd 2   H   Ueq(max)/Ueq(min) Range       4.4 Ratio
PLAT234_ALERT_4_C Large Hirshfeld Difference U06    --  C79A    ..      0.17 Ang.
PLAT244_ALERT_4_C Low    'Solvent' Ueq as Compared to Neighbors of      C1SS Check
PLAT911_ALERT_3_C Missing # FCF Refl Between THmin & STh/L=  0.600        21 Report
PLAT971_ALERT_2_C Check Calcd Residual Density  2.35A From     C00      2.12 eA-3
PLAT971_ALERT_2_C Check Calcd Residual Density  1.39A From     C00      1.85 eA-3
PLAT978_ALERT_2_C Number C-C Bonds with Positive Residual Density.         0 Info
```

Alert level G

```
PLAT002_ALERT_2_G Number of Distance or Angle Restraints on AtSite         6 Note
PLAT003_ALERT_2_G Number of Uiso or Uij Restrained non-H Atoms ...        86 Report
PLAT042_ALERT_1_G Calc. and Reported MoietyFormula Strings  Differ    Please Check
PLAT068_ALERT_1_G Reported F000 Differs from Calcd (or Missing)...    Please Check
PLAT083_ALERT_2_G SHELXL Second Parameter in WGHT  Unusually Large      6.04 Why ?
PLAT176_ALERT_4_G The CIF-Embedded .res File Contains SADI Records         1 Report
PLAT178_ALERT_4_G The CIF-Embedded .res File Contains SIMU Records         3 Report
PLAT186_ALERT_4_G The CIF-Embedded .res File Contains ISOR Records         2 Report
PLAT232_ALERT_2_G Hirshfeld Test Diff (M-X)  U03    --  C20A    ..       9.0 s.u.
PLAT232_ALERT_2_G Hirshfeld Test Diff (M-X)  U04    --  C72A    ..      10.0 s.u.
PLAT232_ALERT_2_G Hirshfeld Test Diff (M-X)  U04    --  C74A    ..       6.0 s.u.
PLAT232_ALERT_2_G Hirshfeld Test Diff (M-X)  U04    --  C77A    ..       5.2 s.u.
PLAT232_ALERT_2_G Hirshfeld Test Diff (M-X)  U04    --  C80A    ..      14.0 s.u.
PLAT232_ALERT_2_G Hirshfeld Test Diff (M-X)  U05    --  C01A    ..       5.4 s.u.
PLAT232_ALERT_2_G Hirshfeld Test Diff (M-X)  U05    --  C02A    ..       6.0 s.u.
PLAT232_ALERT_2_G Hirshfeld Test Diff (M-X)  U05    --  C10A    ..       8.2 s.u.
PLAT232_ALERT_2_G Hirshfeld Test Diff (M-X)  U05    --  C11A    ..       9.8 s.u.
PLAT232_ALERT_2_G Hirshfeld Test Diff (M-X)  U05    --  C13A    ..       6.8 s.u.
PLAT232_ALERT_2_G Hirshfeld Test Diff (M-X)  U06    --  C61A    ..      15.5 s.u.
PLAT232_ALERT_2_G Hirshfeld Test Diff (M-X)  U06    --  C62A    ..      12.5 s.u.
PLAT232_ALERT_2_G Hirshfeld Test Diff (M-X)  U06    --  C65A    ..       6.7 s.u.
PLAT232_ALERT_2_G Hirshfeld Test Diff (M-X)  U06    --  C76A    ..       8.2 s.u.
PLAT232_ALERT_2_G Hirshfeld Test Diff (M-X)  U06    --  C77A    ..       6.8 s.u.
PLAT232_ALERT_2_G Hirshfeld Test Diff (M-X)  U06    --  C78A    ..       7.2 s.u.
PLAT232_ALERT_2_G Hirshfeld Test Diff (M-X)  U06    --  C80A    ..      17.5 s.u.
PLAT301_ALERT_3_G Main Residue  Disorder ..............(Resd  1)..        2% Note
PLAT333_ALERT_2_G Check Large Av C6-Ring C-C Dist. C02A   -C12A         1.43 Ang.
PLAT333_ALERT_2_G Check Large Av C6-Ring C-C Dist. C04A   -C17A         1.43 Ang.
PLAT333_ALERT_2_G Check Large Av C6-Ring C-C Dist. C07A   -C22A         1.42 Ang.
PLAT333_ALERT_2_G Check Large Av C6-Ring C-C Dist. C08A   -C25A         1.43 Ang.
PLAT333_ALERT_2_G Check Large Av C6-Ring C-C Dist. C13A   -C31A         1.42 Ang.
PLAT333_ALERT_2_G Check Large Av C6-Ring C-C Dist. C14A   -C34A         1.43 Ang.
PLAT333_ALERT_2_G Check Large Av C6-Ring C-C Dist. C16A   -C36A         1.42 Ang.
PLAT343_ALERT_2_G Unusual sp?      Angle Range in Main Residue for       C00 Check
PLAT343_ALERT_2_G Unusual     sp?  Angle Range in Main Residue for      C01A Check
PLAT343_ALERT_2_G Unusual     sp?  Angle Range in Main Residue for      C06A Check
PLAT343_ALERT_2_G Unusual     sp?  Angle Range in Main Residue for      C70A Check
PLAT343_ALERT_2_G Unusual     sp?  Angle Range in Main Residue for      C73A Check
PLAT343_ALERT_2_G Unusual     sp?  Angle Range in Main Residue for      C77A Check
PLAT343_ALERT_2_G Unusual     sp?  Angle Range in Main Residue for      C78A Check
PLAT343_ALERT_2_G Unusual     sp?  Angle Range in Main Residue for      C80A Check
PLAT432_ALERT_2_G Short Inter X...Y Contact  N03    ..  C09A    ..      3.01 Ang.
PLAT720_ALERT_4_G Number of Unusual/Non-Standard Labels ..........        44 Note
PLAT802_ALERT_4_G CIF Input Record(s) with more than 80 Characters         1 Info
PLAT860_ALERT_3_G Number of Least-Squares Restraints .............       784 Note
PLAT912_ALERT_4_G Missing # of FCF Reflections Above STh/L=  0.600       104 Note
PLAT913_ALERT_3_G Missing # of Very Strong Reflections in FCF ....         1 Note
```

```
PLAT933_ALERT_2_G Number of OMIT Records in Embedded .res File ...         12 Note
```

```
  6 ALERT level A = Most likely a serious problem - resolve or explain
  1 ALERT level B = A potentially serious problem, consider carefully
 21 ALERT level C = Check. Ensure it is not caused by an omission or oversight
 48 ALERT level G = General information/check it is not something unexpected

  3 ALERT type 1 CIF construction/syntax error, inconsistent or missing data
 59 ALERT type 2 Indicator that the structure model may be wrong or deficient
  5 ALERT type 3 Indicator that the structure quality may be low
  9 ALERT type 4 Improvement, methodology, query or suggestion
  0 ALERT type 5 Informative message, check
```

It is advisable to attempt to resolve as many as possible of the alerts in all categories. Often the minor alerts point to easily fixed oversights, errors and omissions in your CIF or refinement strategy, so attention to these fine details can be worthwhile. In order to resolve some of the more serious problems it may be necessary to carry out additional measurements or structure refinements. However, the purpose of your study may justify the reported deviations and the more serious of these should normally be commented upon in the discussion or experimental section of a paper or in the "special_details" fields of the CIF. checkCIF was carefully designed to identify outliers and unusual parameters, but every test has its limitations and alerts that are not important in a particular case may appear. Conversely, the absence of alerts does not guarantee there are no aspects of the results needing attention. It is up to the individual to critically assess their own results and, if necessary, seek expert advice.

Publication of your CIF in IUCr journals

A basic structural check has been run on your CIF. These basic checks will be run on all CIFs submitted for publication in IUCr journals (*Acta Crystallographica*, *Journal of Applied Crystallography*, *Journal of Synchrotron Radiation*); however, if you intend to submit to *Acta Crystallographica Section C* or *E* or *IUCrData*, you should make sure that full publication checks are run on the final version of your CIF prior to submission.

Publication of your CIF in other journals

Please refer to the *Notes for Authors* of the relevant journal for any special instructions relating to CIF submission.

PLATON version of 13/08/2017; check.def file version of 27/07/2017

Datablock shelx - ellipsoid plot

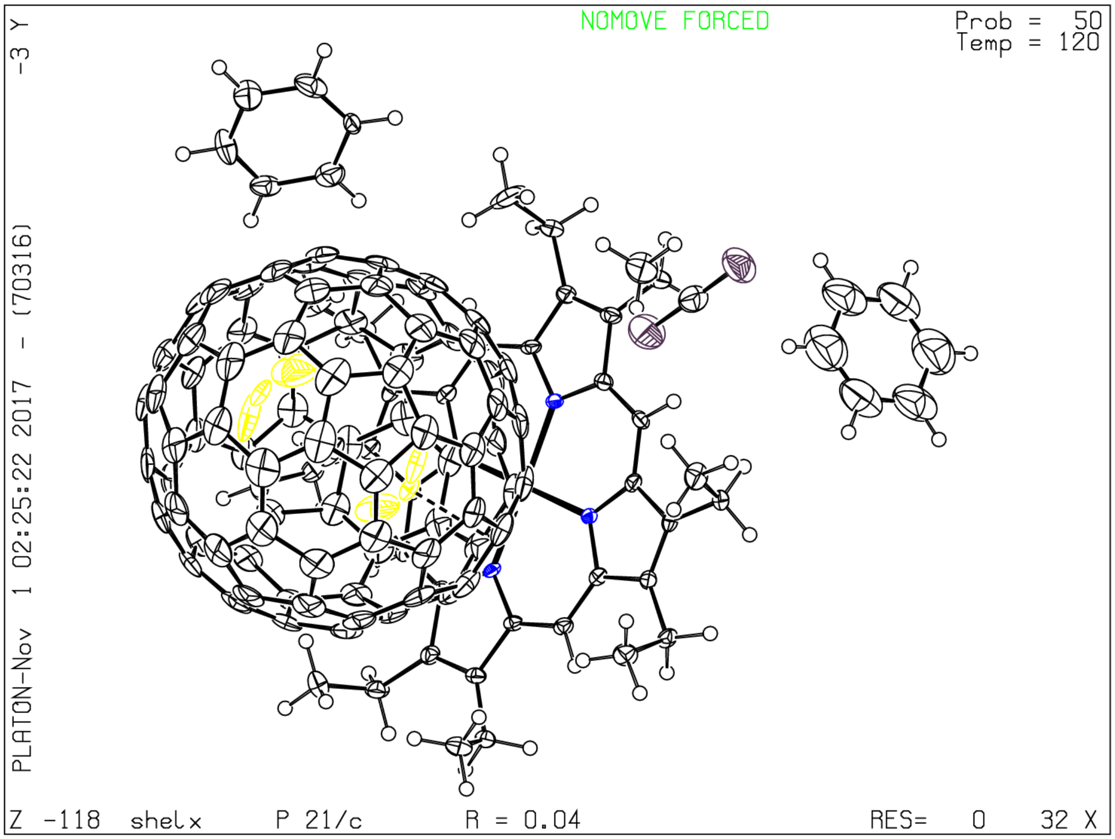

Supplement: Supplementary file 5 — Supplementary Data 2 [file 41467_2018_5210_MOESM5_ESM.pdf]
